# Genome-wide scan for selection signatures reveals novel insights into the adaptive capacity in local North African cattle

**DOI:** 10.1038/s41598-020-76576-3

**Published:** 2020-11-10

**Authors:** Slim Ben-Jemaa, Salvatore Mastrangelo, Seung-Hwan Lee, Jun Heon Lee, Mekki Boussaha

**Affiliations:** 1 Laboratoire des Productions Animales et Fourragères, Institut National de la Recherche Agronomique de Tunisie, Université de Carthage, 2049 Ariana, Tunisia; 2 Dipartimento Scienze Agrarie, Alimentari e Forestali, University of Palermo, 90128 Palermo, Italy; 3 Division of Animal and Dairy Science, Chungnam National University, Daejeon, Korea; 4 Université Paris-Saclay, INRAE, AgroParisTech, GABI, 78350 Jouy-en-Josas, France

**Keywords:** Evolution, Genetics, Molecular biology

## Abstract

Natural-driven selection is supposed to have left detectable signatures on the genome of North African cattle which are often characterized by the fixation of genetic variants associated with traits under selection pressure and/or an outstanding genetic differentiation with other populations at particular loci. Here, we investigate the population genetic structure and we provide a first outline of potential selection signatures in North African cattle using single nucleotide polymorphism genotyping data. After comparing our data to African, European and indicine cattle populations, we identified 36 genomic regions using three extended haplotype homozygosity statistics and 92 outlier markers based on Bayescan test. The 13 outlier windows detected by at least two approaches, harboured genes (e.g. *GH1, ACE, ASIC3, HSPH1, MVD, BCL2*, *HIGD2A*, *CBFA2T3*) that may be involved in physiological adaptations required to cope with environmental stressors that are typical of the North African area such as infectious diseases, extended drought periods, scarce food supply, oxygen scarcity in the mountainous areas and high-intensity solar radiation. Our data also point to candidate genes involved in transcriptional regulation suggesting that regulatory elements had also a prominent role in North African cattle response to environmental constraints. Our study yields novel insights into the unique adaptive capacity in these endangered populations emphasizing the need for the use of whole genome sequence data to gain a better understanding of the underlying molecular mechanisms.

## Introduction

Taurine cattle were first introduced to Africa through Egypt from the Fertile Crescent ~ 6500 years BP^1^ and dispersed into North Africa where they have undergone hybridization with local wild aurochs^2^. The geographic proximity of North Africa to Europe makes it a likely contact zone between the two continents. Several genetic studies reported an old presence of African cattle ancestry in the genomes of Iberian cattle^2,3^ and a European ancestry in local Maghreb cattle^4–6^. Nomad pastoralism and tribal migrations prevented the division of North African cattle populations into clearly defined breed groups. Present-day indigenous cattle in Morocco, Algeria Tunisia and Libya belong to the Brown Atlas cattle. These are small-sized, sturdy, fairly compact animals with fine limbs, a short head and a straight to slightly concave profile. In these countries, Brown Atlas cattle populations, predominantly pasture-fed, are raised in a Mediterranean climate characterized by a winter rainfall and a hot dry summer during which live weight losses in adult cows can reach 20%^7^. In Egypt, indigenous cattle are medium sized, long-bodied animals, lean of musculature and lightly boned. They are raised either in desert or semi-desert regions characterized by a very arid Mediterranean climate and negligible rainfall. A number of ecotypes are recognised based on their geographical distribution. For instance, in Lower Egypt there are two local cattle populations, the Damietta is typically found in coastal sites and the Baladi or Baheri is widespread inland in the delta^8^. Overall, North African indigenous cattle are resistant to many of the diseases and parasites to which imported European cattle are susceptible^7^ resulting from a local environment-driven selection that occurred over hundreds of years. Adaptation to local conditions is expected to leave distinct signatures in the genome known as a “selective sweeps” owing to a rapid increase in the frequency of the desirable alleles or in the frequency of neutral markers in linkage disequilibrium with the favorable alleles^9^. Studies on signatures of selection focusing exclusively on North African cattle have never been reported before.

The emergence of high-throughput single nucleotide polymorphism (SNP) genotyping and whole genome sequencing facilities coupled with the development of new genomic methodologies have enabled the screening of a large part of the genome to detect signatures of selection in livestock and domestic populations^10–14^. All these studies have used comparison of genomic patterns of SNPs variability between local and exotic breeds to identify genomic regions and genes that have undergone selective sweeps.

The main goal of this study was to investigate population structure and candidate positive selection signatures in North African cattle using genotype data from the Illumina BovineSNP50 BeadChip with comparisons against four European breeds, three African and two indicine populations. We applied four genome scan approaches to identify genomic regions putatively under selection: the first three methods are extended haplotype homozygosity (EHH)-derived statistics (*iHS*, *Rsb* and *XP-EHH*) and are based on the decay of haplotype homozygosity as a function of recombination distance. The fourth approach is a Bayesian method based on the differentiation of allele frequencies among populations.

## Results

### Population structure analysis among all cattle populations

**Figure 1:**
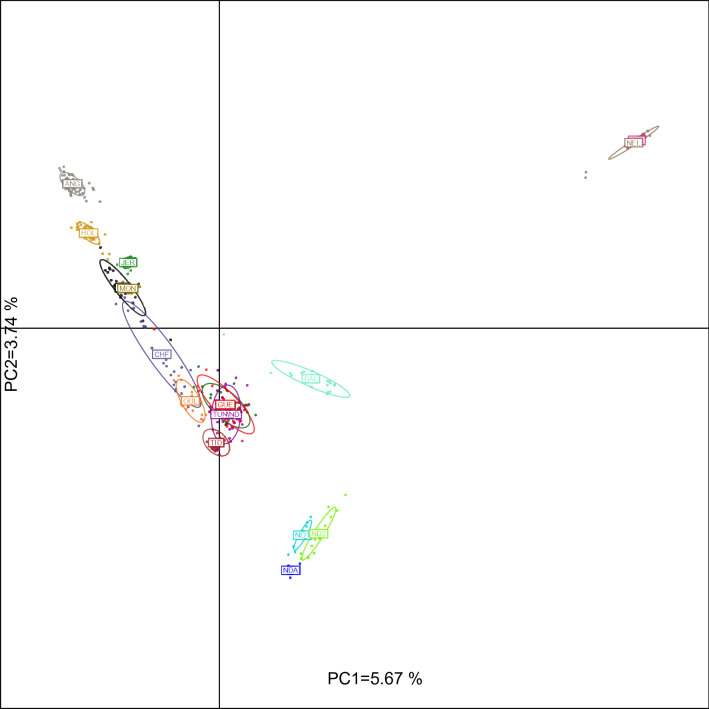
Principle component analysis results of allele frequencies obtained from 38,464 SNPs genotyped in 468 cattle individuals from 17 populations. Each point represents the eigenvalues of principal components 1 and 2. Populations are represented by coloured inertia ellipses.

We used Principal Component Analysis (PCA) to contextualize the genetic variation of North African cattle populations (Fig. 1). The first two principal components accounted for 5.67% (PC1) and 3.74% (PC2) of the total genetic variation. The global organization of the genetic diversity of the populations of the study might be described as a triangle with apexes corresponding to North European breeds (Angus (ANG) and Holstein (HOL)), African taurines (NDA, ND1 and ND2) and indicine populations (NEL and GIR). PCA results show that the Tunisian Brune de l’Atlas (TUNIND) and the Algerian populations (Guelmoise (GUE) and Cheurfa (CHE)) are closer to each other than to the Moroccan (Oulmes Zaer (OUL) and Tidili (TID)) and the Egyptian (Baladi (BAL)) populations. Furthermore, these results distinguished Biskra (BIS) and Chelifienne (CHF) from the other North African populations. The former was positioned near European breeds with several BIS individuals clustering along with Montbéliarde (MON) while CHF individuals showed a higher dispersion around their center of gravity (with several individuals positioned near MON) indicating a high genetic heterogeneity.

**Figure 2:**
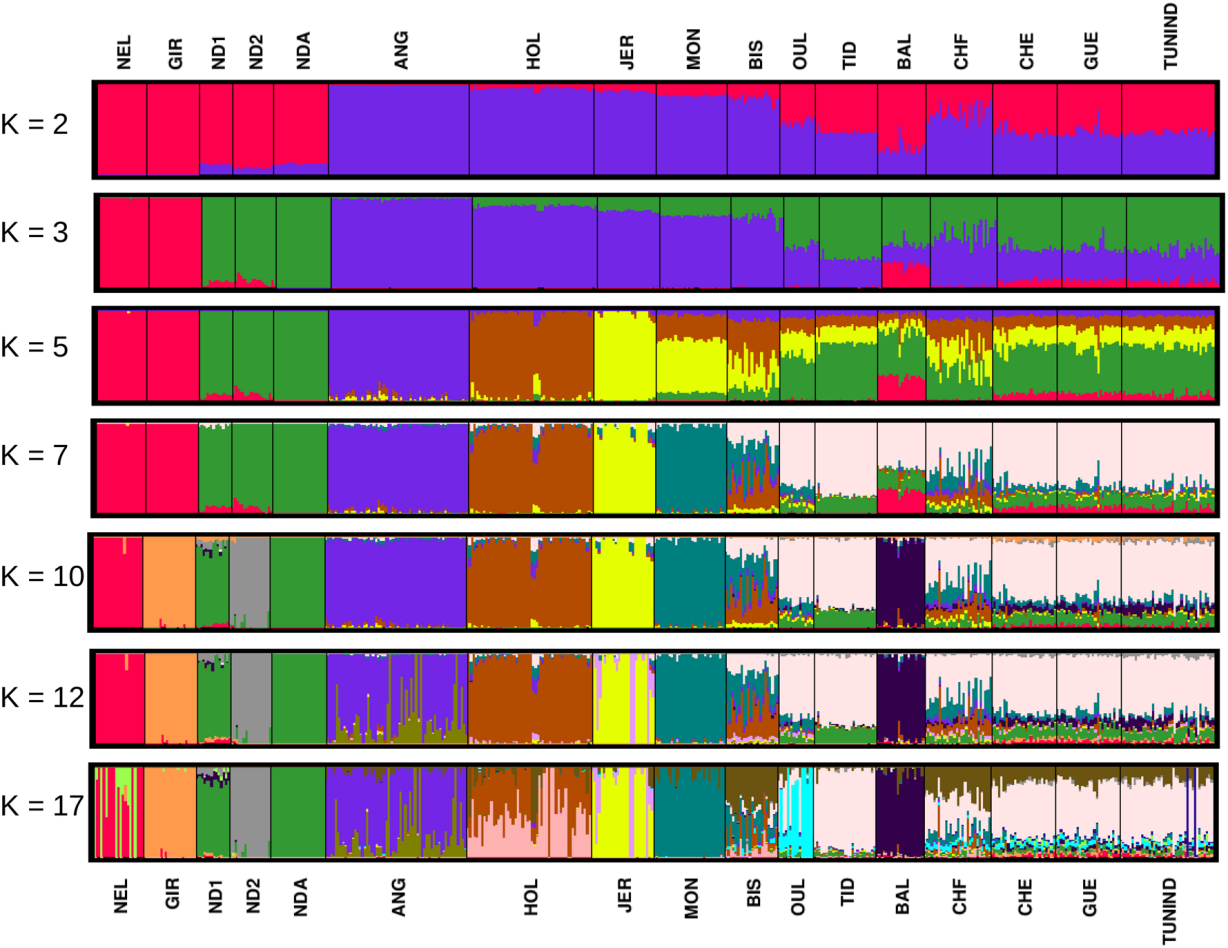
Unsupervised hierarchical clustering of the 468 individuals from the 17 populations of the study. Results for K (number of clusters) = 2, 3, 5, 7, 10 (k-value with the lowest cross-validation error), 12 and 17 are shown. Individuals are grouped by population. Each individual is represented by a vertical bar. The proportion of the bar in each of K colours corresponds to the average posterior likelihood that the individual is assigned to the cluster indicated by that colour. Populations are separated by vertical black lines.

Breed assignment to clusters using ADMIXTURE provided further insight into the genetic structure of North African populations. Figure 2 shows the results obtained for K values 2, 3, 5, 7, 10, 12 and 17. K = 10 showed the lowest cross-validation error (Supplementary Fig. S1). At K = 2, European taurine breeds were separated from indicine and African cattle. The K = 3 model further separated African populations from indicine cattle. All North African populations except BAL carry two main European and African ancestries. In agreement with PCA results, BIS shows the largest amount of European ancestry with a minimum of 61.86% and a maximum of 88.5% while the Moroccan TID has the largest amount of African ancestry with a minimum of 55.67% and a maximum of 70.32%. For its part, BAL possesses a significant amount of indicine ancestry with a minimum of 16.41% and a maximum of 30.35%. At K = 5, the three European breeds (ANG, HOL and Jersey (JER)), formed three different clusters. All North African populations had on average 21.69% (with a minimum of 10.93% in BAL and a maximum of 29.42% in BIS) and 19.47% (with a minimum of 10.85% in BAL and a maximum of 46.37% in BIS) of JER and HOL ancestries, respectively. At K = 7, all North African populations except BIS and a few CHF individuals can be seen as distinct from the other breeds with a major “North African” component ranging, on average, from 48.8% for BAL and CHF to 79.5% for TID. It is worth noting that BIS displayed a substantial level of MON introgression (on average, 32.1%) while no African ancestry was detected within this breed (Fig. 2). At K = 10, BAL separated from the other North African populations while this happened for OUL when K was set to 17.

Details of the level of pairwise genetic differentiation are reported in Supplementary Table S1. Most of North African populations showed low differentiation levels. The lowest *F*_*ST*_ values are found between CHE and GUE (*F*_*ST*_ ~ 0), CHE and TUNIND (*F*_*ST*_ = 0.002) and between GUE and TUNIND (*F*_*ST*_ = 0.003). Likewise, low genetic differentiation is observed between TID on one hand, GUE, CHE and TUNIND, on the other hand (*F*_*ST*_ TID/GUE = 0.016, F_ST_ TID/CHE = 0.016 and F_ST_ TID/TUNIND = 0.015) while a higher F_ST_ is observed between these three breeds and BAL (0.042, 0.042 and 0.045 for BAL/CHE, BAL/GUE and BAL/TUNIND, respectively).

**Figure 3:**
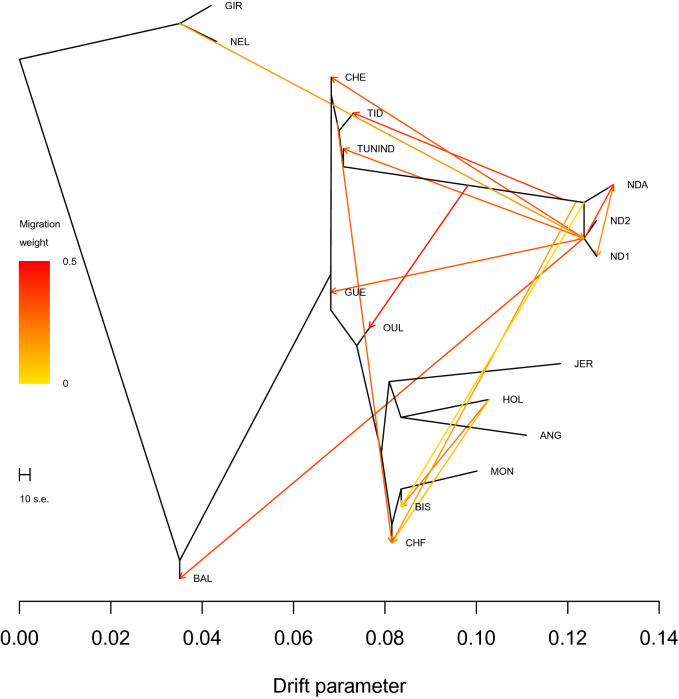
Maximum likelihood tree constructed with TreeMix when 14 migration events (modeled as arrows) were allowed. Migration arrows are coloured according to their weight.

We used the TreeMix software to model both population splits and gene flow between the 17 cattle populations. When no migration events were fit (Supplementary Fig. S2, residuals presented in Supplementary Fig. S3), the eight North African populations were positioned on different locations on the tree. BAL was the closest to indicine populations while BIS was in clade with the European breeds. We then sequentially added migration events to the tree until the proportion of the variance in relatedness between populations explained by the model began to asymptote. This happened when 14 migration edges were fit (where 99.93% of the variance in ancestry between populations was explained by the model (Supplementary Fig. S4)). The phylogenetic network structure presented in Fig. 3 highlights the known African taurine introgression into North African populations and significant levels of admixture from Holstein (HOL) into the genomes of BIS and CHF.

### Candidate genome regions putatively under selection in North African cattle

In order to perform an accurate search for signatures of selection in North African cattle, we selected the breeds that are most representative of the ancestral North African populations i.e. those with a major “North African” component. This was done based on the population structure results and led to the exclusion of BIS (because of the low portion of its North African ancestry) and CHF (because of its high inter-individual genomic heterogeneity) (Figs. 1, 2, 3). We also removed a total of 1475 SNPs because of uncertainty in the identification of their ancestral state (see methods section).

*Rsb* and Cross-population Extended Haplotype Homozygosity (*XP-EHH)* statistics were computed at each SNP for each of the three comparisons (African (AFT)/North African, European (EUT)/North African, indicine (IND)/North African). Haplotypes estimated in each population were pooled, for each autosome, according to their group of origin. In total, 108, 334 and 86 haplotypes were considered as representative of African, European, and indicine ancestries, respectively.

#### EHH-based methods

**Figure 4:**
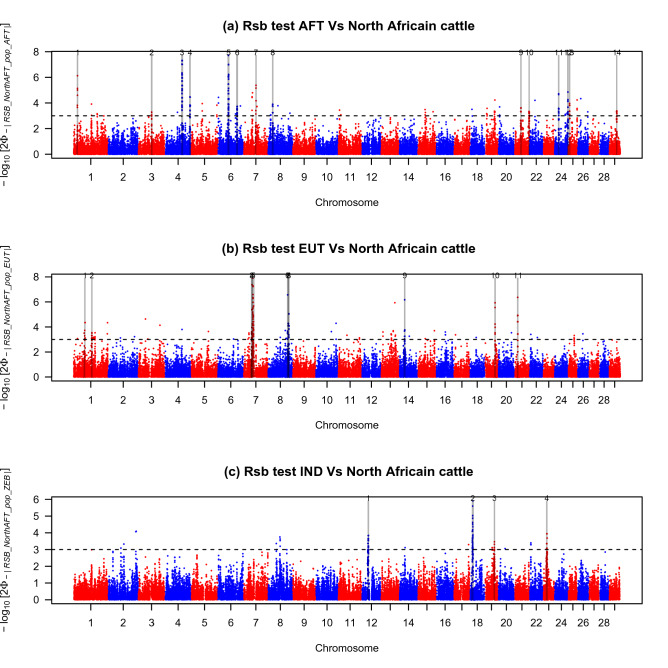
Manhattan plots showing the results of *Rsb* test for the autosomes in North African cattle. (**a**) Rsb test AFT versus North African cattle. (**b**) Rsb test EUT versus North African cattle. (**c**) Rsb test IND versus North African cattle. Horizontal dashed lines mark the significance threshold applied to detect the outlier SNPs (–log10 (*p* value) = 3).

**Figure 5:**
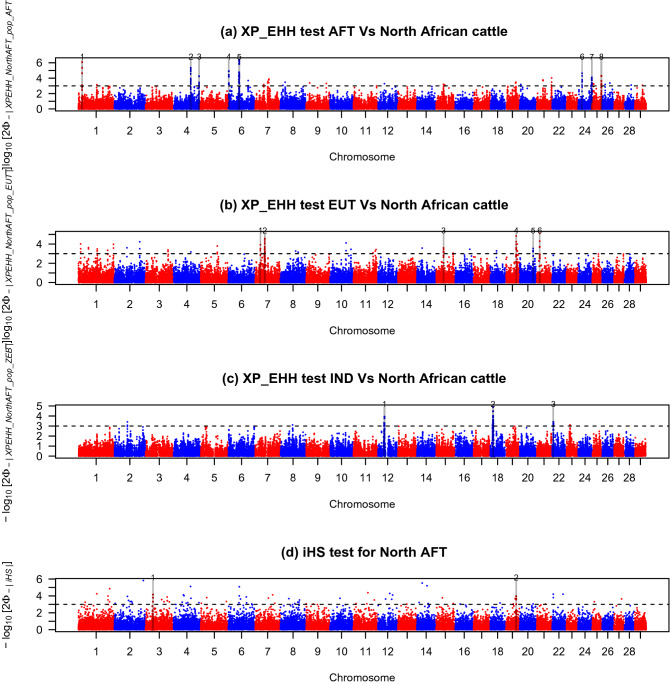
Manhattan plots showing the results of *XP-EHH* and *iHS* tests for the autosomes in North African cattle. (**a**) *XP-EHH* test AFT versus North African cattle. (**b**) *XP-EHH* test EUT versus North African cattle. (**c**) *XP-EHH* test IND versus North African cattle. (**d**) *iHS* test for North African cattle. Horizontal dashed lines mark the significance threshold applied to detect the outlier SNPs (–log10 (*p* value) = 3).

*Rsb* detected 427, 369 and 167 SNPs putatively under selection for AFT/North AFT, EUT/North AFT and IND/North AFT comparisons, respectively (Fig. 4a–c, respectively). These markers defined 14, 11 and 4 candidate regions for the comparisons between North AFT and AFT, North AFT and EUT and North AFT and IND, respectively (Fig. 4, Table 1). *XP-EHH* yielded fewer outlier SNPs than analyses based on the *Rsb* approach: 254, 196 and 111 SNPs putatively under selection for AFT/North AFT, EUT/North AFT and IND/North AFT comparisons, respectively (Fig. 5a–c, respectively). These outliers defined 8, 6 and 3 selective sweeps for the comparisons between North AFT and AFT, North AFT and EUT and North AFT and IND, respectively (Table 1). Among these, six, three and two regions were also identified with Rsb tests for AFT/North AFT, EUT/North AFT and IND/North AFT comparisons, respectively (Table 1). These regions are located on chromosomes (BTA) 01 (at position: 17,740,000–19,640,000 bp), BTA04 (at positions: 76,470,000–78,910,000 bp and 113,060,000–114,940,000 bp), BTA06 (at position: 46,780,000–50,050,000 bp) and BTA24 (at positions :18,030,000–20,020,000 bp and 59,750,000–61,740,000 bp) for the AFT/North AFT comparison, on BTA07 (at position: 41,060,000–43,620,000 bp), BTA19 (at position: 47,120,000–49,070,000 bp) and BTA21 (at position: 14,830,000–16,650,000 bp) for the EUT/North AFT comparison and on BTA12 (at position: 28,400,000–30,490,000 bp), BTA18 (at position: 11,580,000–14,350,000 bp) for the IND/North AFT comparison. The intra-population *iHS* analysis revealed a total of 2 significant regions (p*iHS* ≥ 3) distributed on BTA 03 (at position: 32,200,000–33,750,000) and 19 (at position: 47,390,000–48,980,000) (Fig. 5d, Table 1). The latter region was also revealed by the EUT/North African comparison (both *Rsb* and *XP-EHH* tests). Overall, the 11 candidate genomic regions identified by at least two EHH-based methods, overlap with Quantitative Trait Loci (QTL) associated with traits for milk and meat composition, fertility and sexual precociousness, disease susceptibility (tuberculosis and respiratory diseases), stature and growth (Supplementary Table S2). Also, the 11 aforementioned genomic regions co-localize with 166 previously described structural variants most of which (148 out of 166) are copy number variations (CNV) (Supplementary Table S3). In total, 71 genes are located in CNV regions (Supplementary Table S4).

**Table 1:** Genomic regions putatively under selection identified using *iHS*, *Rsb *and* XP-EHH *statistics. Regions jointly identified by at least two methods are in bold.

Test	BTA	Start (pb)	End (pb)	length (Mb)	Genes
iHS	3	32,200,000	33,750,000	1.55	DENND2D, CEPT1, DRAM2, LRIF1, U6, CD53, KCNA3, KCNA2, KCNA10, CYM, PROK1, LAMTOR5, SLC16A4, RBM15, KCNC4, SLC6A17, bta-mir-2285as-3, UBL4B, ALX3, STRIP1, AHCYL1, CSF1, bta-mir-2413, EPS8L3, GSTM3, GSTM1
	**19**	**47,390,000**	**48,980,000**	**1.59**	**TANC2, CYB561, ACE, KCNH6, DCAF7, TACO1, MAP3K3, LIMD2, STRADA, CCDC47, DDX42, FTSJ3, PSMC5, SMARCD2, bta-mir-10173, TCAM1, GH1, CD79B, SCN4A, ERN1, SNORD104, SNORA50C, TEX2, PECAM1, MILR1, POLG2, DDX5, bta-mir-3064, CEP95, SMURF2, KPNA2**
RsbAFT versus North AFT	**1**	**17,680,000**	**19,640,000**	**1.96**	**U6, 5S_rRNA, 5S_rRNA, TMPRSS15, CHODL, C1H21orf91, U6, BTG3, CXADR, 7SK**
	3	59,870,000	61,700,000	1.83	PRKACB, TTLL7, 5S_rRNA
	**4**	**76,470,000**	**78,910,000**	**2.44**	**RAMP3, TBRG4, SNORA5C, SNORA5A, CCM2, SNORA9, MYO1G, U6, PURB, bta-mir-4657, H2AFV, PPIA, ZMIZ2, OGDH, TMED4, DDX56, NPC1L1, NUDCD3, CAMK2B, YKT6, GCK, MYL7, POLD2, AEBP1, POLM, BLVRA, COA1, STK17A, HECW1, MRPL32, PSMA2, C4H7orf25, GLI3**
	**4**	**113,060,000**	**114,940,000**	**1.88**	**GIMAP4, GIMAP7, GIMAP5, TMEM176B, 5S_rRNA, TMEM176A, AOC1, KCNH2, NOS3, ATG9B, ABCB8, ASIC3, CDK5, SLC4A2, FASTK, bta-mir-6525, TMUB1, AGAP3, ASB10, GBX1, IQCA1L, ABCF2, CHPF2, bta-mir-671, SMARCD3, NUB1, WDR86, CRYGN, RHEB, PRKAG2, GALNTL5, GALNT11, KMT2C, CCT8L2**
	**6**	**46,780,000**	**50,050,000**	**3.27**	**5S_rRNA, SNORA70, Y_RNA, U6, PCDH7**
	6	86,650,000	88,640,000	1.99	SLC4A4, GC, NPFFR2, ADAMTS3, SNORD42, COX18, ANKRD17, ALB, AFP, AFM, RASSF6
	7	54,670,000	56,540,000	1.87	YIPF5, KCTD16, U6
	8	20,180,000	22,020,000	1.84	ELAVL2, DMRTA1, bta-mir-2285cd
	21	29,320,000	31,200,000	1.88	CHRNA7, U6, OTUD7A, ADAMTS7, TBC1D2B, SH2D7, CIB2, IDH3A, ACSBG1, DNAJA4, WDR61, CRABP1, IREB2, HYKK, PSMA4, CHRNA5, CHRNA3, CHRNB4, UBE2Q2, FBXO22
	21	66,740,000	68,120,000	1.38	bta-mir-1247, DIO3, PPP2R5C, U6, DYNC1H1, HSP90AA1, WDR20, MOK, ZNF839, CINP, U5, TECPR2, ANKRD9, RCOR1, TRAF3, AMN, CDC42BPB, EXOC3L4, 5S_rRNA, TNFAIP2, EIF5, SNORA28, MARK3
	**24**	**18,030,000**	**20,020,000**	**1.99**	**CELF4**
	**24**	**59,660,000**	**61,790,000**	**2.13**	**CDH20, RNF152, PIGN, RELCH, SNORD36, TNFRSF11A, ZCCHC2, PHLPP1, BCL2, KDSR, U6, VPS4B, SERPINB5**
	25	5,540,000	7,420,000	1.88	RBFOX1, U2
	29	33,910,000	35,070,000	1.16	OPCML, NTM
Rsb EUT versus North AFT	1	51,390,000	52,980,000	1.59	bta-mir-2286, CCDC54, 5S_rRNA, BBX, CD47, IFT57
	1	82,820,000	84,650,000	1.83	THPO, POLR2H, CLCN2, FAM131A, EIF4G1, SNORD66, PSMD2, ECE2, CAMK2N2, ALG3, VWA5B2, bta-mir-1224, ABCF3, AP2M1, DVL3, EIF2B5, HTR3C, ABCC5, PARL, MAP6D1, YEATS2, KLHL24, KLHL6, SNORA63, MCF2L2, B3GNT5, LAMP3, MCCC1, DCUN1D1, ATP11B
	7	34,800,000	36,670,000	1.87	DTWD2
	**7**	**36,720,000**	**38,670,000**	**1.95**	**SEMA6A, 5S_rRNA, COMMD10, ARL10, NOP16, HIGD2A, CLTB, FAF2, RNF44, CDHR2, GPRIN1, SNCB, EIF4E1B, TSPAN17, UNC5A, HK3, UIMC1, ZNF346, U6, FGFR4, NSD1**
	**7**	**39,450,000**	**46,350,000**	**6.9**	**CLK4, 7SK, ZNF354A, PROP1, 5S_rRNA, OR2Y1, MGAT1, ZFP62, BTNL9, OR2V1, TRIM7, TRIM41, RACK1, SNORD96, TRIM52, IFI47, ZNF496, U6, NLRP3, OR2B11, GCSAML, OR2G2, OR2G3, OR6F1, OR11L1, TRIM58, OR2AJ1, OR2L13, OR2M4, OR2T6, OR2T1, OR2T27, OR2T11, OR2G6, MGC137030, LYPD8, SH3BP5L, ZNF672, ZNF692, PGBD2, PLPP2, MIER2, THEG, C2CD4C, SHC2, ODF3L2, MADCAM1, TPGS1, CDC34, GZMM, BSG, HCN2, POLRMT, FGF22, RNF126, FSTL3, PRSS57, PALM, MISP, PTBP1, PLPPR3, AZU1, PRTN3, ELANE, CFD, MED16, R3HDM4, KISS1R, ARID3A, WDR18, GRIN3B, TMEM259, CNN2, ABCA7, ARHGAP45, POLR2E, GPX4, SBNO2, STK11, CBARP, ATP5F1D, MIDN, CIRBP, FAM174C, EFNA2, PWWP3A, NDUFS7, GAMT, DAZAP1, RPS15, APC2, C7H19orf25, PCSK4, REEP6, ADAMTSL5, MEX3D, MBD3, UQCR11, TCF3, ONECUT3, ATP8B3, REXO1, KLF16, ABHD17A, SCAMP4, CSNK1G2, bta-mir-6120, BTBD2, SOWAHA, SHROOM1, GDF9, UQCRQ, LEAP2, AFF4, ZCCHC10, HSPA4, FSTL4, C7H5orf15, VDAC1, TCF7, SKP1, PPP2CA, bta-mir-2285di, CDKL3, UBE2B, CDKN2AIPNL, JADE2, SAR1B, SEC24A, CAMLG, DDX46, C7H5orf24**
	**8**	**88,100,000**	**90,020,000**	**1.92**	**5S_rRNA, GADD45G, SEMA4D, SECISBP2, CKS2, SHC3, S1PR3, Vault, 5S_rRNA, NXNL2, SPIN1**
	8	91,150,000	92,450,000	1.3	ALDOB, TMEM246, RNF20, GRIN3A
	8	93600000	95530000	1.93	SMC2, OR13C3, OR13C8, NIPSNAP3A, ABCA1, SLC44A1, FSD1L, FKTN, TAL2, TMEM38B
	14	23,020,000	24,720,000	1.7	TMEM68, TGS1, LYN, RPS20, U1, MOS, PLAG1, CHCHD7, SDR16C5, SDR16C6, PENK, U6, IMPAD1, FAM110B, UBXN2B, CYP7A1, U1
	**19**	**47,120,000**	**49,070,000**	**1.95**	**MARCHF10, TANC2, CYB561, ACE3, ACE, KCNH6, DCAF7, TACO1, MAP3K3, LIMD2, STRADA, CCDC47, DDX42, FTSJ3, PSMC5, SMARCD2, bta-mir-10173, TCAM1, GH1, CD79B, SCN4A, ERN1, SNORD104, SNORA50C, TEX2, PECAM1, MILR1, POLG2, DDX5, bta-mir-3064, CEP95, SMURF2, KPNA2, C17orf58, BPTF**
	**21**	**14,830,000**	**16,650,000**	**1.82**	**SLCO3A1, SV2B, U6, AKAP13, KLHL25**
Rsb IND versus North AFT	**12**	**28,400,000**	**30,490,000**	**2.09**	**PDS5B, N4BP2L2, N4BP2L1, BRCA2, ZAR1L, FRY, RXFP2, bta-mir-2299, B3GLCT, HSPH1, TEX26, MEDAG, ALOX5AP, USPL1, HMGB1, KATNAL1**
	**18**	**10,590,000**	**15,060,000**	**4.47**	**ATP2C2, MEAK7, COTL1, KLHL36, USP10, CRISPLD2, ZDHHC7, KIAA0513, FAM92B, GSE1, GINS2, EMC8, COX4I1, IRF8, FOXF1, MTHFSD, FOXC2, FOXL1, FBXO31, MAP1LC3B, ZCCHC14, JPH3, KLHDC4, SLC7A5, CA5A, BANP, ZNF469, ZFPM1, ZC3H18, CYBA, MVD, SNAI3, CTU2, RNF166, PIEZO1, bta-mir-2327, CDT1, APRT, GALNS, TRAPPC2L, CBFA2T3, ACSF3, CDH15, SLC22A31, ANKRD11, SPG7, RPL13, SNORD68, CPNE7, DPEP1, CHMP1A, CDK10, SPATA2L, VPS9D1, ZNF276, FANCA, SPIRE2, TCF25, MC1R, TUBB3, DEF8, DBNDD1, GAS8, U1, SHCBP1, VPS35, ORC6, MYLK3**
	19	44,340,000	45,970,000	1.63	MEIOC, CCDC43, DBF4B, ADAM11, GJC1, HIGD1B, EFTUD2, bta-mir-2343, CCDC103, FAM187A, GFAP, KIF18B, C1QL1, DCAKD, NMT1, PLCD3, ACBD4, HEXIM1, HEXIM2, FMNL1, MAP3K14, U6, ARHGAP27, PLEKHM1, RDM1, LYZL6, RPRML, GOSR2, WNT9B, WNT3, NSF, ARF2, CRHR1, SPPL2C, MAPT
	23	16,430,000	18,420,000	1.99	BICRAL, RPL7L1, PTCRA, CNPY3, GNMT, PEX6, PPP2R5D, MEA1, KLHDC3, RRP36, CUL7, MRPL2, KLC4, PTK7, SRF, CUL9, DNPH1, TTBK1, SLC22A7, CRIP3, U6, ZNF318, ABCC10, DLK2, TJAP1, LRRC73, YIPF3, POLR1C, XPO5, POLH, GTPBP2, MAD2L1BP, RSPH9, MRPS18A, VEGFA, U6, TMEM63B, CAPN11, MYMX, SLC29A1, HSP90AB1, SLC35B2, NFKBIE, TMEM151B, AARS2, SPATS1, CDC5L, SUPT3H, 5S_rRNA
XP-EHH AFT versus North AFT	**1**	**17,740,000**	**19,640,000**	**1.9**	**U6, 5S_rRNA, TMPRSS15, CHODL, C1H21orf91, BTG3, CXADR, 7SK**
	**4**	**76,570,000**	**78,910,000**	**2.34**	**CCM2, SNORA9, MYO1G, U6, PURB, bta-mir-4657, H2AFV, PPIA, ZMIZ2, OGDH, TMED4, DDX56, NPC1L1, NUDCD3, CAMK2B, YKT6, GCK, MYL7, POLD2, AEBP1, POLM, BLVRA, COA1, STK17A, HECW1, MRPL32, PSMA2, C4H7orf25, GLI3**
	**4**	**113,110,000**	**114,860,000**	**1.75**	**GIMAP4, GIMAP7, GIMAP5, TMEM176B, 5S_rRNA, TMEM176A, AOC1, KCNH2, NOS3, ATG9B, ABCB8, ASIC3, CDK5, SLC4A2, FASTK, bta-mir-6525, TMUB1, AGAP3, ASB10, GBX1, IQCA1L, H2BE1, ABCF2, CHPF2, bta-mir-671, SMARCD3, NUB1, WDR86, CRYGN, RHEB, PRKAG2, GALNTL5, GALNT11, KMT2C**
	6	2,080,000	4,020,000	1.94	MARCHF1, TMA16, TKTL2, NPY5R, NPY1R, NAF1, U6, BBS7, CCNA2, EXOSC9, ANXA5, U3
	**6**	**46,780,000**	**50,050,000**	**3.27**	**5S_rRNA, SNORA70, Y_RNA, PCDH7**
	**24**	**18,030,000**	**20,020,000**	**1.99**	**CELF4**
	**24**	**59,750,000**	**61,740,000**	**1.99**	**CDH20, RNF152, PIGN, RELCH, SNORD36, TNFRSF11A, ZCCHC2, PHLPP1, BCL2, KDSR, U6, VPS4B, SERPINB5**
	25	39,370,000	41,300,000	1.93	SDK1, bta-mir-2390, CARD11, GNA12, AMZ1, BRAT1, bta-mir-11980, IQCE, TTYH3, LFNG, bta-mir-12029, GRIFIN, CHST12, bta-mir-12019, EIF3B, SNX8, NUDT1, MRM2, MAD1L1, ELFN1
XP-EHH EUT versus North AFT	7	22,580,000	24,390,000	1.81	FNIP1, U6, bta-mir-12018, 7SK, CDC42SE2, LYRM7, HINT1, CHSY3, MINAR2
	**7**	**41,060,000**	**43,620,000**	**2.56**	**OR6F1,OR11L1, TRIM58, OR2W3, 5S_rRNA, OR2AJ1, OR2L13, OR2M4, OR2T6, OR2T1, OR2T27, OR2T11, OR2G6, U6, MGC137030, LYPD8, SH3BP5L, ZNF672, ZNF692, PGBD2, PLPP2, MIER2, THEG, C2CD4C, SHC2, ODF3L2, MADCAM1, TPGS1, CDC34, GZMM, BSG, HCN2, POLRMT, FGF22, RNF126, FSTL3, PRSS57, PALM, MISP, PTBP1, PLPPR3, AZU1, PRTN3, ELANE, CFD, MED16, R3HDM4, KISS1R, ARID3A, WDR18, GRIN3B, TMEM259, CNN2, ABCA7, ARHGAP45, POLR2E, GPX4, SBNO2**
	15	33,830,000	35,760,000	1.93	GRAMD1B, SCN3B, ZNF202, SAAL1, TPH1, SERGEF, KCNC1, MYOD1, OTOG, USH1C, ABCC8, KCNJ11, NCR3LG1, NUCB2, PIK3C2A, RPS13, SNORD14, PLEKHA7, U6, C15H11orf58
	**19**	**47,120,000**	**49,140,000**	**2.02**	**MARCHF10, TANC2, CYB561, ACE, KCNH6, DCAF7, TACO1, MAP3K3, LIMD2, STRADA, CCDC47, DDX42, FTSJ3, PSMC5, SMARCD2, bta-mir-10173, TCAM1, GH1, CD79B, SCN4A, ERN1, SNORD104, SNORA50C, TEX2, PECAM1, MILR1, POLG2, DDX5, bta-mir-3064, CEP95, SMURF2, KPNA2, C17orf58, BPTF**
	20	57,910,000	59,770,000	1.86	U6, ANKH, OTULIN, OTULINL, TRIO, DNAH5
	**21**	**14,800,000**	**16,630,000**	**1.83**	**SLCO3A1, SV2B, U6, AKAP13, KLHL25**
XP-EHH IND versus North AFT	**12**	**28,400,000**	**30,490,000**	**2.09**	**PDS5B, N4BP2L2, N4BP2L1, BRCA2, ZAR1L, FRY, RXFP2, bta-mir-2299, B3GLCT, HSPH1, TEX26, MEDAG, ALOX5AP, USPL1, HMGB1, KATNAL1**
	**18**	**11,580,000**	**14,350,000**	**2.77**	**GSE1, GINS2, EMC8, COX4I1, IRF8, FOXF1, MTHFSD, FOXC2, FOXL1, FBXO31, MAP1LC3B, ZCCHC14, JPH3, KLHDC4, SLC7A5, CA5A, BANP, ZNF469, ZFPM1, ZC3H18, CYBA, MVD, SNAI3, CTU2, RNF166, PIEZO1, bta-mir-2327, CDT1, APRT, GALNS, TRAPPC2L, CBFA2T3, ACSF3, CDH15, SLC22A31, ANKRD11**
	22	4,920,000	6,550,000	1.63	TGFBR2, GADL1, U6, STT3B, OSBPL10

#### Bayesian F_ST_ method

We used the BayeScan program to identify putative genomic regions under selection in North African cattle. A total of 53 and 39 outlier SNPs were detected for *F*_ST_ AFT/North AFT and *F*_ST_ EUT/North AFT, respectively (Supplementary Fig. S5, Supplementary Tables S5, S6). Among these 92 SNPs, only five markers were located within or close to candidate regions detected by an EHH-based metric (Supplementary Tables S5, S6). No significant SNPs were identified with the *F*_ST_ IND/North AFT test.

### Identification and functional annotation of the genes within the candidate regions

Outlier windows from *iHS*, *Rsb* and *XP-EHH* tests include 57, 581 and 305 known genes, respectively (Table 1). Genes identified with *Rsb* and *XP-EHH* are distributed as follows: 151 and121, 264 and 127, 166 and 57 for AFT/North African, EUT/North African, IND/North African comparisons, respectively (Table 1). Thirty genes were common to both *iHS* and EUT/North African comparison (either *Rsb* or *XP-EHH*). Similarly, 109, 143 and 65 genes were jointly identified by *Rsb* and *XP-EHH* for each of the AFT/North African, EUT/North African, IND/North African comparisons, respectively of which 74, 97 and 50, respectively, could be mapped by DAVID Bioinformatics resources (https://david.ncifcrf.gov/). Gene Ontology (GO) analysis showed that AIG1 (IPR006703, n = 6, Benjamini-corrected *p* value = 4.45 × 10^−7^) and P-loop containing nucleoside triphosphate hydrolase (IPR027417, n = 14, Benjamini-corrected *p* value  = 0.0031) InterPro protein functional groups were the two significantly enriched functional classes identified in the AFT/North AFT comparison (Supplementary Table S7). Sensory perception of smell (GO:0,007,608, n = 18, Benjamini-corrected *p* value = 3.23 × 10^−14^) and G-protein coupled receptor signaling pathway (GO:0,007,186, n = 19, Benjamini-corrected *p* value  = 3.48 × 10^−6^) were the most enriched biological process (BP) terms identified in the EUT/North AFT comparison. Olfactory receptor activity (GO:0,004,984, n = 21, Benjamini-corrected *p* value  = 6.66 × 10^−7^) and serine-type endopeptidase activity (GO:0,004,252, n = 6, Benjamini-corrected *p* value  = 0.053) were the most enriched terms under molecular function (MF) in the same comparison (Supplementary Table S8).

## Discussion

The main purpose of the present study is to unravel signatures of positive selection in North African cattle. Because we used several breeds with diverse population structure, the main challenge in our study was to minimize the rate of false-positive signals that can arise, inter alia, owing to the confounding effects of population demographics^15^. Assuming that populations with similar structure have undergone similar evolutionary processes, in our selection signature detection analyses, we retained only North African populations showing a high degree of within population genetic homogeneity and a large portion of North African ancestry. In agreement with previous studies^6^ our genome analyses are consistently and strongly in the direction of a substantial and recent contribution of European breeds to the genomes of BIS and CHF (Figs. 1, 2). Furthermore, in the admixture models in which K = 7, 10 and 12, the individuals sampled from these two breeds showed a high degree of within population genetic heterogeneity. Therefore, BIS and CHF were discarded from the subsequent selection signature analyses.

Our results corroborate previous reports^16^ suggesting that BAL resulted from a three-way admixture between breeds representative of European, African and indicine cattle. The presence of an indicine content within the genome of BAL is consistent with a wave of indicine introduction during the rinderpest epidemic of the nineteenth century^1,17^. Our results indicate that all North African populations share ancestry with Jersey cattle which supports previous whole genome sequencing analyses reporting a common distinct patriline of Jersey bulls with African cattle^18^. Overall, our findings indicate that modern North African cattle can be classified into 3 subgroups. The first one is the “Brune de l’Atlas” population which possesses two main African and European ancestries. This subgroup includes the Moroccan TID, the Algerian GUE and CHE and the Tunisian Brune de l’Atlas. The second subgroup consists of the Egyptian local cattle which possesses an additional large portion of indicine ancestry (at the expense of European ancestry). The third subgroup, represented by CHF and BIS, includes European-derived breeds. The phylogenetic network inferred by TreeMix corroborate these findings in that CHF and especially BIS are in clade with the European breeds while CHE, TID, TUNIND and GUE share the same branch and are much closer to African populations.

In this paper, we present the first genome-wide scan of putative selective sweeps in North African cattle by combining four different statistical methods based either on the decay of haplotype homozygosity as a function of recombination distance or on allele frequency differentiation among populations. In total, we highlight the presence of 36 different genomic regions putatively under selection using the first type of approaches (*iHS, Rsb* and *XP-EHH*) and 92 outlier SNPs using Bayescan. Consistently with previous observations^19^, we observe little overlap between results obtained from each of the two types of approaches. Given that Bayescan assumes that the gene frequencies under any neutrally structured population model can be approximated by a multinomial Dirichlet distribution^20^ which would not be appropriate in a hierarchical population structure^21^ (as is the case for our North African sample), the 92 identified SNPs should be considered cautiously. Instead, we believe that the three EHH-based methods, which inter alia, can detect a wider range of selection scenarios^22^, are more suitable to our study design. These statistics take advantage of the reduction in haplotype diversity in the neighbourhood of a beneficial mutation due to a “hitch-hiking” effect. They measure the extended haplotype homozygosity which is defined as the probability of identity by descent for two randomly chosen haplotypes carrying a core haplotype of interest in an interval around a given locus, given that they have the same allele at the locus^23^. Unlike *Rsb* and *XP-EHH,* the *iHS* test has low power in identifying fixed sweeps because it requires the ancestral allele to be still segregating in the population^24^. Here, we identified a higher number of outlier windows using *Rsb* and *XP-EHH* compared to the *iHS* approach which might suggest, at first glance, that most of the candidate regions identified here have undergone a positive selection resulting in the (near) fixation of the favoured alleles across the populations. However, we believe that the low number of candidate regions identified by the *iHS* test is actually due to the fact that this approach searches for loci where a given high-frequency haplotype is much longer relative to all other haplotypes, yet in a soft sweep several long haplotypes will be present at the adaptive locus and thus not one haplotype will typically be much longer than all others^25^. Our hypothesis assumes that the majority of sweeps detected here are soft which is likely to be the case. Soft sweeps were shown to be widespread and account for the vast majority of recent environmental adaptation in several species such as Humans^24^. A common constraint of selection signature detection methods is the detection of false positives. One efficient way to reduce their number is to retain as outliers, those genomic regions detected by distinct methods^26^. Among the 36 genomic regions identified by EHH-based methods, 10 were detected by two tests and one candidate region was identified by all three tests. In addition, two other regions (BTA07: 36,720,000–38,670,000 bp and BTA08: 88,100,000–90,020,000 bp) identified by the *Rsb* EUT/North AFT comparison included two outlier SNPs detected by Bayescan. We particularly focused on genes located within these 13 genomic regions. In agreement with previous findings^27,28^, we observed that the three candidate regions jointly identified by the *Rsb* and *XP-EHH* tests in the EUT/North African comparison were significantly enriched for genes involved in olfactory receptor activity (21 genes) which might reflect the fact that selection has been relaxed around these genes in European breeds which are often raised in abundant food supply conditions. Two genes (*OR2W3* and *OR2L13*) coincided with CNVs previously reported in cattle (Supplementary Table S4). Olfactory receptor genes are duplicated within the bovine genome^27^ and CNVs encompassing these genes were found to be associated with population-specific differences in smell in most mammalian species^29^.

Many of our candidate regions harboured genes implicated in the adaptive immune response against microbial pathogens. For instance, the clearest sweep signal in the EUT/North AFT comparison detected on BTA07 (between positions: 41.06 and 43.62 Mb) with 13 SNPs (out of 32) exceeding the significance threshold, harboured 58 known genes amongst which six (*AZU1*, *ELANE*, *GZMM*, *PRSS57, PRTN3, CFD*) belong to the S1A family of peptidases, a superfamily of proteolytic enzymes with a wide variety of biological functions in parasite infection^30^. Similarly, another relevant selection signature on the BTA19 jointly detected by *iHS*, *Rsb* and *XP-EHH* EUT/North African harboured several genes which are involved in immune response: *CD79B*, *MILR1, PECAM1, MAP3K3* and *TCAM1*. The last two genes mediate NF-kappa-B activity which show evidence of positive selection in the African N’Dama cattle to alter in functions to effectively regulate the infection of cattle trypanosome^31^. Consistently, we also observed that outlier windows from AFT/North African and IND/North African comparisons included many genes associated with immune response and host defence such as *TNFRSF11A*, *IRF8*, *MYO1G* and several GTPases of immunity-associated protein (GIMAP) genes (GIMAP4, GIMAP5 and GIMAP7). Several of these genes (GIMAP4, GIMAP5, GIMAP7, *IRF8*) coincided with CNVs reported in cattle (Supplementary Table S4). A major phenotype of North African cattle populations is their resistance to parasitic diseases such as theileriosis, babesiosis and anaplasmosis^32^ which are highly prevalent in North Africa^33^. We suggest that the aforementioned genes have been under evolutionary pressure in North African cattle and that some of them may have experienced enhanced fixation of duplicates resulting from selection for increased dosage to effectively regulate the innate and acquired immune response to parasitic diseases. A previous study^34^ conducted on Brazilian *Bos indicus* cattle, similarly reported that CNVs are important modulators of immune gene expression. Our results have also revealed a series of other genes involved in the regulation of blood pressure and heart contraction (*ACE*, *ACE3*, *COX4I1*, *NOS3, CXADR*), blood vessel development and morphogenesis (*CCM2*, *FOXC2*, *FOXF1*, *MAP3K3*). These genes are expected to be involved in adaptation to extreme temperatures prevailing in several Northern African areas and/or to chronic hypoxia in the Atlas mountain ranges where the altitude varies between 900 and 4000 m^7^. Our hypothesis is consistent with the presence of three hypoxia-related genes (*BCL2*, *HIGD2A* and *CBFA2T3*) and three other genes involved in response to heat (*ASIC3*, *HSPH1* and *MVD)* in the relevant candidate regions (Table 1). It is also interesting to note that the strong selection signal on BTA19 harboured a well-known gene, *GH1,* linked to response to nutrient levels (GO: 0031667), positive regulation of lactation (GO:1903489) and triglyceride biosynthetic process (GO:0010867) and was previously reported as being a candidate gene for dairy production traits in Braunvieh cattle^15^. Importantly, it has been suggested that elevated *GH1* gene expression may constitute an adaptive response to the effects of scarce food supply in a sample of 163 human individuals from Benin^35^. We therefore suggest that this gene is particularly under positive selection across North African cattle populations as a consequence of important seasonal fluctuations in food availability characterizing the whole region.

Six out of the 13 relevant candidate regions identified in this study, harboured fewer than 15 known protein coding genes (Table 1). Many of these genes have also been reported in cattle and other species. For instance, the outlier window on BTA01 (at position: 17,740,000–19,640,000 bp), contained 6 protein coding genes including *TMPRSS15* and *CHODL*, two genes that were reported to be under selection in the Iraqi indigenous cattle^13^. Similarly, the candidate region on the BTA24 (at position: 59,660,000–61,790,000 bp), harboured *RNF152* gene which positively regulates Toll-like receptors (TLRs) which are important pattern recognition receptors that are critical for the defence against invading pathogens^36^. *RNF152* gene was reported to be involved in local adaptations in the Ainu, a hunter-gatherer population of northern Japan^37^. Another relevant candidate region on BTA21 (at position: 14,830,000–16,650,000 bp) harboured four protein coding genes: *SLCO3A1, SV2B, AKAP13* and *KLHL25.* The latter two genes were shown to be under positive selection in Creole cattle breeds^38^ while *SLCO3A1* is associated with marbling score in the Montana Tropical Composite beef cattle^39^ and mediates inflammatory processes in intestinal epithelial cells through NF-kappa-B transcription activation in humans^40^. *SV2B* gene is among major genes enriched for the extracellular matrix (ECM) around the hair follicle in Changthangi goats^41^. ECM is considered important for regulating the structure, metabolism and signaling of dermal papilla cells which play key roles in hair follicle morphogenesis and regeneration^42^. Another candidate region on the BTA22 (at position: 4,790,000–6,620,000 bp) identified by the *XP-EHH* IND versus North AFT test harboured four genes (*GADL1, TGFBR2*, *STT3B* and *OSBPL10)* and among these, *GADL1* gene is one of the genes involved in adaptive evolution of *Anolis carolinensis* introduced into the Ogasawara archipelago^43^. Gadl1^−/−^ mice exhibited decreased anxiety, increased levels of oxidative stress markers, alterations in energy and lipid metabolism, and age-related changes^44^. *STT3B* is a catalytic subunit of hetero oligomeric oligosaccharyltransferase (OST), which is important for asparagine linked glycosylation. In mammals and plants, OSTs exhibit distinct levels of enzymatic efficiency or different responses to stressors^45^. *OSBPL10* gene confers African-ancestry protection against dengue haemorrhagic fever in admixed Cubans^46^.

A further result is that the 13 outlier windows identified by at least two approaches included myriad of genes involved in transcriptional regulation (*AEBP1, ARID3A, BANP, CBFA2T3, DDX5, FTSJ3, GLI3, MIER2, POLR2E, POLRMT, FOXC2, FOXF1, FOXL1, SMARCD2, SMARCD3, TNFRSF11A, BPTF, CDK5, …)* as well as many non-coding RNAs including 9 small nucleolar RNAs (snoRNAs), 12 microRNAs (miRNAs), 10 small nuclear RNAs (snRNA) and 13 long noncoding RNAs (lncRNAs). In addition, many of the aforementioned genes (*BANP, CBFA2T3, GLI3, POLR2E, POLRMT, FOXC2, FOXF1, FOXL1*) co-localize with known cattle CNVs. It is worth noting that CNVs encompassing a gene encoding a transcription factor has a greater phenotypic impact because it can affect both the coding sequence of the gene itself as well as the expression of downstream targets of that gene. From a selective standpoint, these findings suggest that natural selection has shaped North African cattle genome not only through variation in coding sequence but also through extensive regulation of gene expression occurring both at the transcriptional and post-transcriptional level. Lending further support to this hypothesis, the relevant candidate region on BTA24 (at position: 59,750,000–61,740,000 bp) harbours a single gene, *CELF4,* coding for an RNA-binding protein mainly expressed in central nervous system that regulates the expression of many genes co-transcriptionally or post-transcriptionally via interactions with mRNA^47^. Celf4-deficient mice have additional neurological abnormalities including hyperactivity and hyperphagia-associated obesity^48^. Similarly, the most relevant selection signal in the AFT/North AFT comparison (BTA06 at position: 46,780,000—50,050,000 bp) harboured one protein coding gene (*PCDH7*) which coincides with a known CNV (Supplementary Table S4), one 5S ribosomal RNA (5S rRNA) and three non-coding RNA genes: SNORA70, Y_RNA and U6 (Table 1). *PCDH7* is one of the key genes involved in oncogenesis and/or differentiation of the cancer stem cells through a change in its histone methylation status^49^. Likewise, 5S rRNA genes are highly methylated in Arabidopsis thaliana and their expression is under epigenetic control^50,51^.

During the process of fixation of adaptive variants, linked neutral markers are dragged along with the selected site; thus reducing the levels of genetic diversity in the region, while simultaneously new mutations accumulate in the region. The initial frequency of these mutations is low, so that a DNA sequence harbouring a positively selected variant will also harbour an excess of rare derived alleles. Bearing this in mind, we expect that many other sweeps are not detected by our genome scan owing to ascertainment schemes used to discover the BovineSNP50 BeadChip. Clearly, shedding light on additional selective sweeps in North African cattle would require the use of whole genome sequence data and the inclusion of all variants in genetic analyses.

The present study highlighted, for the first time, the presence of putative selection signatures in six local North African cattle populations. Information about the location of these regions can now be used as a starting point to identify causal genetic variants that control some environmental adaptation traits in local breeds which can be utilized in the genetic improvement of commonly used commercial breeds world-wide. Our results are unique in indicating that selection have shaped North African cattle genome through extensive regulation of gene expression whereby the individuals get adapted to short as well as long-term environmental changes. Understanding the functional consequences of such adaptive elements remains a challenge to overcome.

## Methods

### Data merging and SNP filtering

We combined Illumina BovineSNP50 BeadChip genotypes of 57 Brune de l’Atlas individuals (TUNIND) sampled from our previously published data^4,52^ with data already available for 221 animals belonging to seven North African populations (BAL, BIS, CHE, CHF, GUE, TID and OUL) obtained from Flori et al*.*^16^ and Gautier et al*.*^53^. We also included genotyping data belonging to 9 other populations, representatives of European taurines (EUT) (four breeds: ANG, HOL, JER and MON), African taurines (AFT) (three N’Dama populations: ND1, ND2 and NDA) and indicine (two populations: GIR and NEL) from Matukumalli et al*.*^54^. All genotypes were recovered from the web-interfaced genetic Diversity Exploration (WIDDE) database^55^. We performed a relatedness test between individuals within each population using PLINK^56^. The software calculates a variable called PI-HAT reflecting extended haplotypes shared between distantly related individuals. For European, indicine and African breeds, we removed closely related individuals if the PI-HAT value was greater than 0.25 which is a value roughly corresponding to relationships closer than grandsire-granddaughter. For the North African populations, in which natural service is commonly used rather than artificial insemination and are thus generally less inbred, we used a more stringent threshold and excluded one individual from each pair of individuals with a PI-HAT value > 0.1. In total, after relatedness filtering, 468 individuals including 204 North African animals, were available for the different analyses (Supplementary Table S10). We also applied a series of quality control procedures to the genotype data. First, we excluded rare SNPs with low minor allele frequencies (MAF) < 0.05. Then, the whole genotype dataset was subjected to linkage disequilibrium (LD) pruning using the default parameters of PLINK (SNP window size:50, step 5 SNPs, r^2^: 0.5). In total, 38,464 SNPs spread over all autosomal chromosomes were finally considered for population structure analyses.

### Population structure and genetic relationship analyses

Population structure was inferred by PCA for African, European, indicine and North African populations using the adegenet R package^57^. Unsupervised hierarchical clustering was carried out for all populations using ADMIXTURE 1.23 software^58^. We ran ADMIXTURE with cross-validation for values of K from 2 through 17 (the number of populations) to identify the best value of K clusters. DISTRUCT software^59^ was then used to graphically display ancestry within each individual. The pairwise fixation index (*F*_*ST*_) between populations was estimated using Genepop 4.6 software^60^. The patterns of population splits and mixtures were inferred using TreeMix^61^. First, we built a maximum likelihood tree of the 17 populations of the study with no migration events allowed and setting GIR as outgroup. Then, we built a phylogenetic tree of these populations and started adding migration events (modeled as edges) sequentially to the phylogenetic model. The migration edges were added until 99.93% of the variance in ancestry between populations was explained by the model. The residuals from the fit of the model to the data were visualized using the R script implemented in TreeMix.

### Identification of selection signatures

To perform selection signature detection, we selected the individuals that are most representative of the ancestral North African cattle. This was done based on the results of model-based clustering results. We used the population differentiation based analysis implemented in BayeScan (*F*_*ST*_)^62^ and three extended haplotype homozygosity (EHH)-based tests (*iHS*, *Rsb* and *XP-EHH*) to detect signatures of selection within North African cattle. Bayescan, *Rsb* and *XP-EHH* analyses were performed for each of the three pairwise comparisons: North African cattle versus AFT, North African cattle versus EUT and North African cattle versus IND. Bayescan uses a reversible-jump Markov Chain Monte Carlo to separate locus-specific effects of selection from population-specific effects of demography. Outliers are those loci that require the locus-specific component to explain observed genetic diversity. For the Markov chain Monte Carlo (MCMC) algorithm we used 20 pilot runs of 5,000 iterations, a burn-in of 50,000 iterations, a thinning interval of 10 (5,000 iterations were used for the estimation of posterior odds) with a resulting total number of 100,000 iterations. To control the number of false positives, significant SNPs were defined by applying a *q*-value threshold of 0.05.

Haplotype extended patterns were investigated using three metrics implemented in *rehh* package^63^: the *iHS* within-population statistic^64^ and two between-population methods: *Rsb*^65^ and *XP-EHH*^66^. In *iHS* computation, the information on the ancestral and derived allele state is needed for each SNP because this statistic is based on the ratio of the EHH associated to each allele. In our analysis, the ancestral allele was inferred as the most common allele within 3 out-group species including yak, buffalo and sheep. *iHS* scores for each SNP were transformed into two-sided *p* values: p*iHS* =  − log10[1–2|Φ(*iHS*)-0.5|]. As a prerequisite to the *Rsb* and *XP-EHH* computation, haplotypes were reconstructed from the genotyped SNPs using fastPHASE 1.4^67^. The following options were used for each chromosome: -T10 -Ku60 -Kl10 -Ki10. Considering that *Rsb* and *XP-EHH* values are normally distributed, a Z-test was applied to identify significant SNPs under selection. Two-sided *p* value s were derived as p*Rsb* =  − log10[1–2|Φ(*Rsb*)-0.5|] and p*XP-EHH* =  − log10[1–2|Φ(*XP-EHH*)-0.5|] where Φ (x) represents the Gaussian cumulative distribution function. In EHH-based tests, the maximum allowed gap between two SNPs was set to 500 Kb. We used 1-Mb sliding windows that partially overlapped 10 kb with adjacent windows to perform selection signature detection. A window is classified as putatively under selection when it contains at least 3, 4 and 4 markers exceeding the significance threshold of − log10 (*p* value) = 3 for *iHS, Rsb* and *XP-EHH* tests, respectively. Finally, we checked the overlap of the candidate genomic regions detected with at least two EHH-based approaches with the previously identified bovine Quantitative Trait Loci (QTL) available in the cattle QTL database (https://www.animalgenome.org/cgi-bin/QTLdb/BT/index). The overlaps were checked using QTL coordinates according to the Bos taurus genome assembly ARS-UCD1.2.

### Gene identification and functional enrichment analysis

Candidate genome region intervals detected by at least two EHH-based methods (*iHS*, *Rsb*, *XP-EHH*) were interrogated for genes annotated to the *Bos taurus* genome assembly ARS-UCD1.2 using BioMart tool of Ensembl (https://www.ensembl.org/biomart/martview/c8fe3a69961a4088a55b7a249db7e2fa). Cattle structural variants which overlapped the genomic coordinates (in bp) of these relevant candidate selective sweep regions were retrieved using the same database. We have only considered structural variants of less than 8 Mb which corresponds to the maximum size that can be identified, from whole genome sequence data, by the pindel software (https://gmt.genome.wustl.edu/packages/pindel/user-manual.html). We used the online tool, Database for Annotation, Visualization and Integrated Discovery (DAVID) software version 6.8 (https://david.ncifcrf.gov/) for functional enrichment analysis of the genes retrieved from BioMart. GO enrichment analysis included two aspects: Biological Process and Molecular Function. For the GO functional groups and InterPro functional terms returned from DAVID functional analysis, we considered an adjusted Benjamini-corrected *p* value threshold of ≤ 0.05.

## Supplementary information

Supplementary Information 1.
