# Supplementary material for: Genome-wide scan for selection signatures reveals novel insights into the adaptive capacity in local North African cattle
Source: Sci Rep. 2020 Nov 10;10:19466. doi: 10.1038/s41598-020-76576-3 (PMC7655849; doi:10.1038/s41598-020-76576-3)
Supplement: Supplementary file 1 — Supplementary Information 1. [file 41598_2020_76576_MOESM1_ESM.pdf]

Genome-wide scan for selection signatures reveals novel insights into the adaptive capacity in local North African cattle

Supplementary material

Slim Ben-Jemaa[1*], Salvatore Mastrangelo[2], Seung-Hwan Lee[3], Jun Heon Lee[3], Mekki Boussaha[4]

[1]Laboratoire des Productions Animales et Fourragères, Institut National de la Recherche Agronomique de Tunisie, Université de Carthage, 2049 Ariana, Tunisia.

[2]Dipartimento Scienze Agrarie, Alimentari e Forestali, University of Palermo, 90128 Palermo, Italy.

[3]Division of Animal and Dairy Science, Chungnam National University, Daejeon, Korea

[4]Université Paris-Saclay, INRA, AgroParisTech, GABI, 78350 Jouy-en-Josas, France.

[*]Corresponding author: Slim Ben-Jemaa

Email: benjemaaslim@gmail.com

Tel: +216-53498579

Supplementary Figure S1. Cross-validation error values in ADMIXTURE analysis with K ranging from 2 to 17.

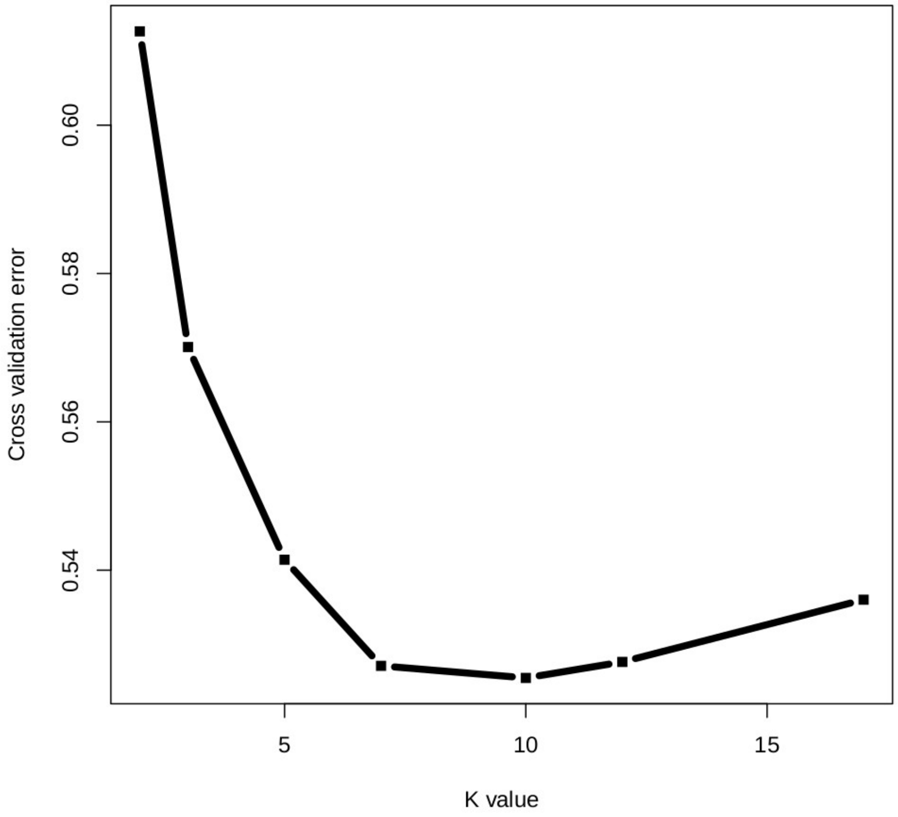

Supplementary Figure S2. Maximum likelihood tree constructed with TreeMix inferred from 17 cattle populations when no migration edges were fit.

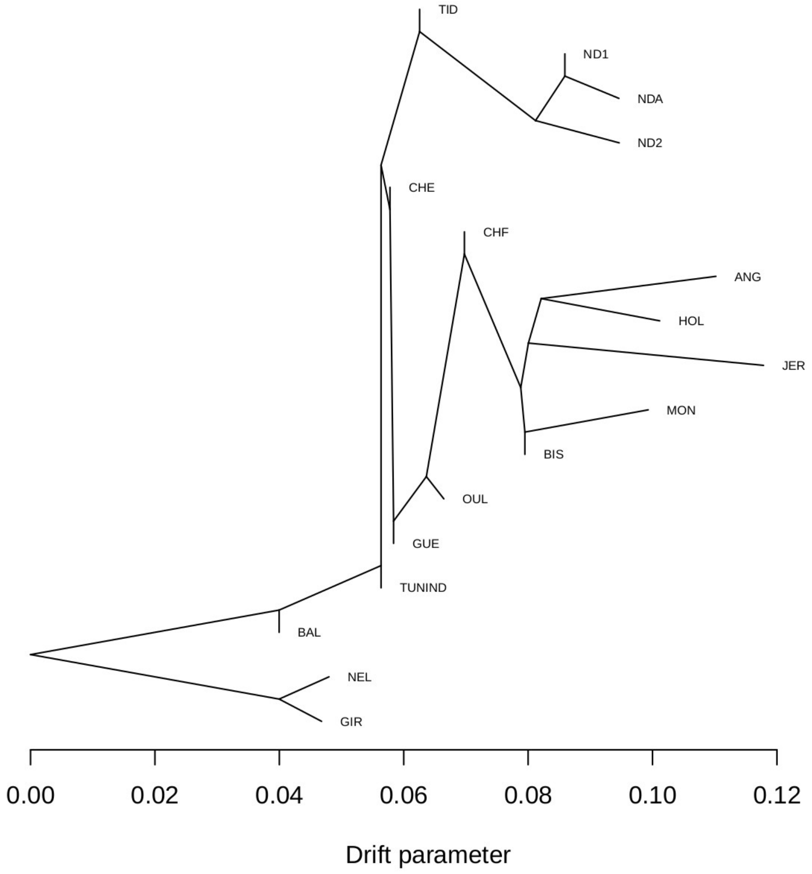

Supplementary Figure S3. Residual Plot from the maximum likelihood tree obtained when no migration edges were fit (Supplementary Figure S2). Colours are described in the palette on the right.

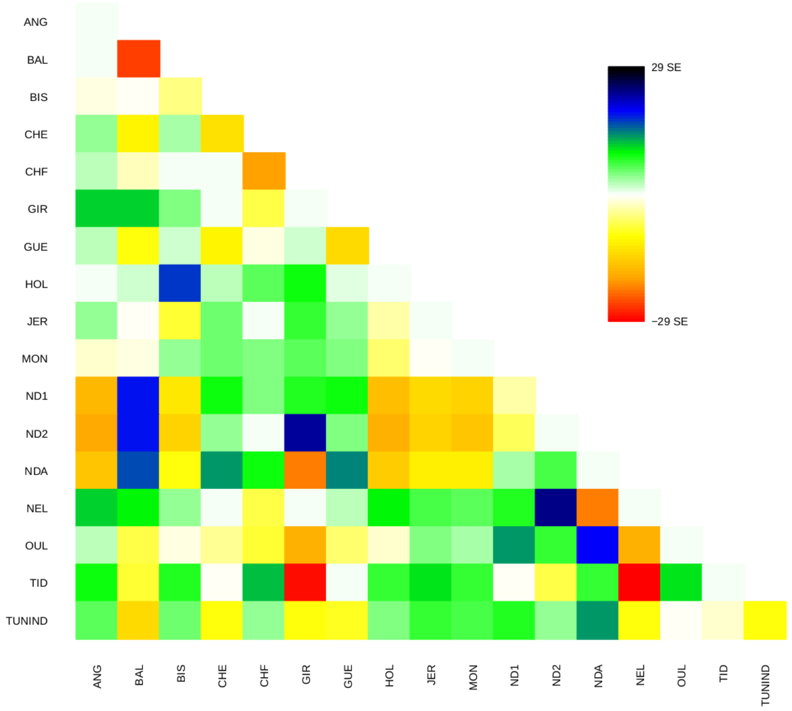

Supplementary Figure S4. The proportion of variance in relatedness between populations explained by phylogenetic models with 0 to 15 migrations.

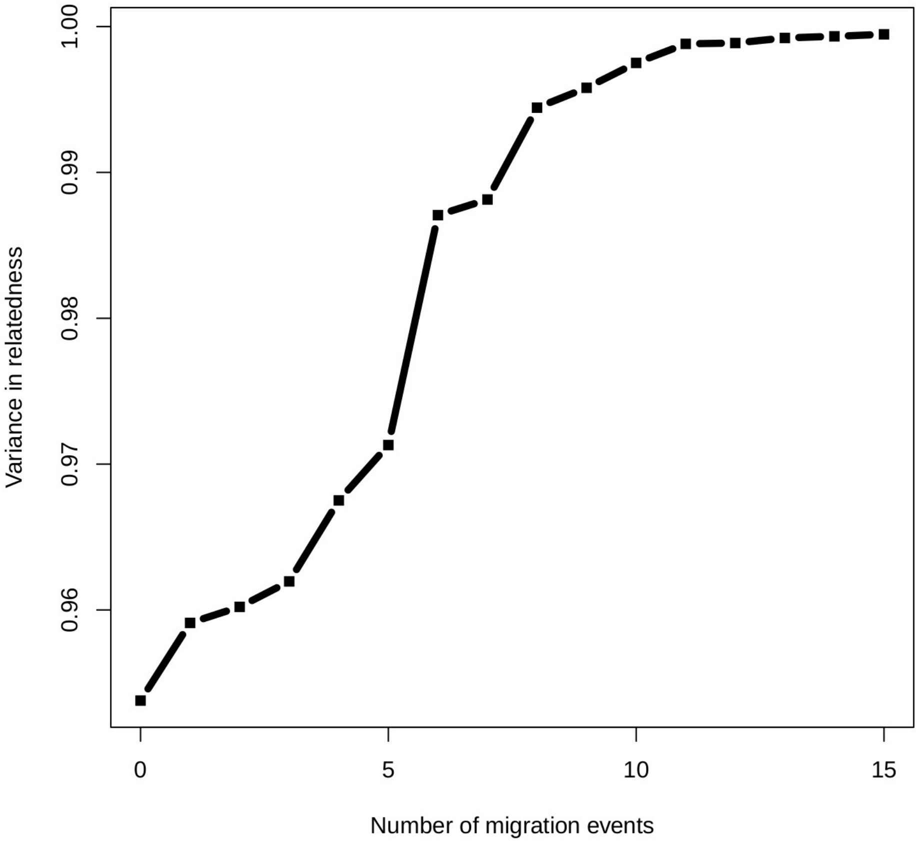

Supplementary Figure S5. Manhattan plot of the pairwise genome-wide autosomal F_{ST} analyses generated by BayeScan in North African cattle. (a) Bayescan EUT/North AFT. (b) Bayescan AFT/North AFT. The red lines indicate the threshold of significance set at 0.05.

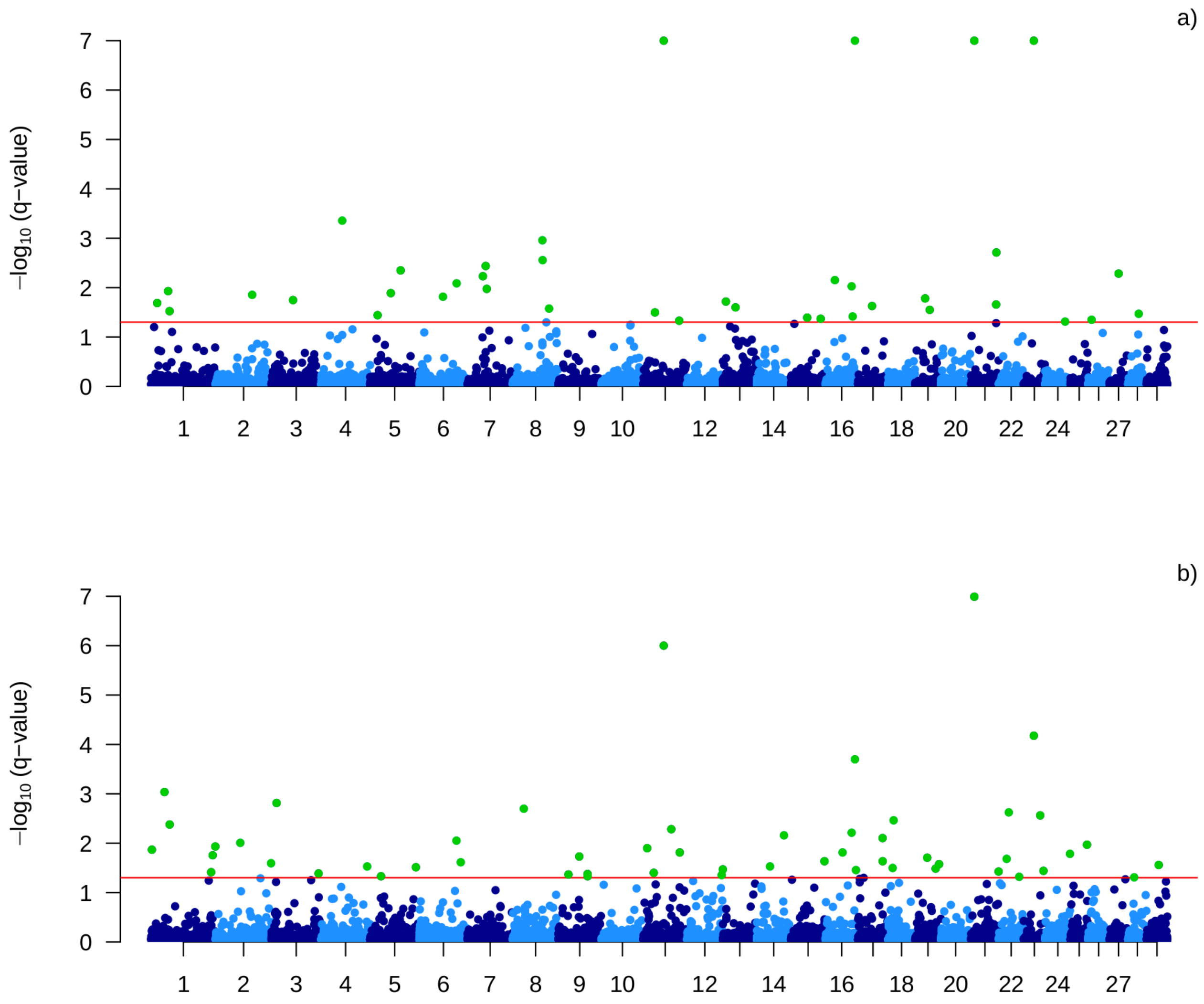

Supplementary Table S1. Pairwise F_{ST} values between cattle populations.

	ANG	BAL	BIS	CHE	CHF	GIR	GUE	HOL	JER	MON	ND1	ND2	NDA	NEL	OUL	TID	TUNIND
ANG																	
BAL	0.171																
BIS	0.090	0.092															
CHE	0.131	0.042	0.047														
CHF	0.098	0.060	0.013	0.013													
GIR	0.353	0.244	0.323	0.284	0.301												
GUE	0.131	0.042	0.047	-0.001	0.014	0.282											
HOL	0.128	0.146	0.042	0.109	0.069	0.338	0.109										
JER	0.179	0.203	0.122	0.155	0.124	0.416	0.155	0.164									
MON	0.145	0.153	0.052	0.105	0.068	0.368	0.104	0.126	0.173								
ND1	0.229	0.098	0.154	0.071	0.101	0.390	0.072	0.207	0.268	0.216							
ND2	0.253	0.131	0.186	0.108	0.136	0.400	0.108	0.232	0.296	0.244	0.082						
NDA	0.254	0.137	0.186	0.098	0.129	0.430	0.099	0.232	0.295	0.244	0.028	0.096					
NEL	0.355	0.249	0.326	0.286	0.304	0.078	0.285	0.340	0.420	0.370	0.394	0.405	0.434				
OUL	0.134	0.073	0.052	0.024	0.024	0.346	0.024	0.114	0.162	0.110	0.104	0.146	0.133	0.349			
TID	0.153	0.063	0.071	0.016	0.030	0.333	0.016	0.130	0.179	0.132	0.073	0.114	0.096	0.337	0.036		
TUNIND	0.134	0.045	0.052	0.002	0.016	0.280	0.003	0.113	0.156	0.109	0.068	0.104	0.094	0.283	0.025	0.015	

Supplementary Table S2. Bovine QTL (http://www.animalgenome.org/cgibin/QTLdb/BT/index) mapped within the candidate genome region intervals identified with at least two EHH-based methods.

Analysis	BTA	Start_interval (bp)	End_interval (bp)	QTL_ID	Start	Stop	Trait
AFT/NorthAFT comparison (*Rsb* and *XP-EHH*)	1	17680000	19640000	-	-	-	-
	4	76470000	78910000	146249	76745254	76745294	Stillbirth (maternal)
	4	76470000	78910000	114139	76840727	76840767	Milk casein percentage
	4	76470000	78910000	114257	76840727	76840767	Milk protein percentage
	4	76470000	78910000	155271	77115576	77115616	Milk caproic acid content
	4	76470000	78910000	155453	77115576	77115616	Milk caprylic acid content
	4	76470000	78910000	169882	77426116	77426156	Sexual precociousness
	4	113060000	114940000	154928	113814159	113814199	Somatic cell count
	4	113060000	114940000	169825	114009663	114009703	Sexual precociousness
	4	113060000	114940000	155289	114414409	114414449	Milk caproic acid content
	4	113060000	114940000	155517	114414409	114414449	Milk caprylic acid content
	4	113060000	114940000	160058	114686662	114686662	Bovine respiratory disease susceptibility
	4	113060000	114940000	31700	114838382	114838422	Average daily gain
	6	46780000	50050000	26754	46834289	46834365	Milk protein percentage
	6	46780000	50050000	26199	46834289	46834365	Milk protein yield
	6	46780000	50050000	25430	46834289	46834365	Milk yield
	6	46780000	50050000	25836	46834289	46834365	Milk fat yield
	6	46780000	50050000	28119	46834289	46834365	Stature
	6	46780000	50050000	152414	48095054	48095094	Saturated fatty acid content
	6	46780000	50050000	67692	48739744	48739784	Body weight gain
	6	46780000	50050000	15944	48940803	51835081	Somatic cell score
	6	46780000	50050000	15945	48940803	51835081	Milk yield
	6	46780000	50050000	177019	49162038	49162078	Conception rate

	6	46780000	50050000	113748	49947893	49947933	Milk kappa-casein percentage
	24	18030000	20020000	155052	18203943	18203983	Milk arachidonic acid content
	24	18030000	20020000	168094	18749086	18749126	Bovine tuberculosis susceptibility
	24	18030000	20020000	178604	19559600	19559640	Meat color
	24	59780000	61840000	151436	60699788	60699828	Abomasum displacement
	24	59780000	61840000	155935	60774400	60774440	tridecylic acid content
EUT/NorthAFT comparison (*Rsb, XP-EHH)* and *iHS*	7	41060000	43620000	167550	41523693	41523733	Rump angle
	7	41060000	43620000	177036	42180767	42180807	Conception rate
	7	41060000	43620000	151496	42904104	42904144	Muscle anserine content
	7	41060000	43620000	151968	42904104	42904144	Muscle potassium content
	7	41060000	43620000	152016	42904104	42904144	Kidney, pelvic, and heart fat percentage
	7	41060000	43620000	152220	42904104	42904144	Muscle sodium content
	7	41060000	43620000	152276	42904104	42904144	Muscle phosphorus content
	7	41060000	43620000	152580	42904104	42904144	Muscle zinc content
	19	47120000	49070000	152298	47402488	47402528	Muscle phosphorus content
	19	47120000	49070000	152149	47631790	47631830	Monounsaturated fatty acid content
	19	47120000	49070000	152374	47631790	47631830	Saturated fatty acid content
	19	47120000	49070000	177228	47668240	47668280	Conception rate
	19	47120000	49070000	10035	47755713	47866768	Milk myristic acid content
	19	47120000	49070000	10037	47755713	47866768	Milk oleic acid content
	19	47120000	49070000	11426	47755713	47866768	Palmitoleic acid content
	19	47120000	49070000	11427	47755713	47866768	Stearic acid content
	19	47120000	49070000	11430	47755713	47866768	Oleic acid content
	19	47120000	49070000	11431	47755713	47866768	Monounsaturated to saturated fatty acid ratio
	19	47120000	49070000	14824	47755713	47866768	Oleic acid content
	19	47120000	49070000	18888	47922295	48033350	Myristic acid content

	19	47120000	49070000	18889	47922295	48033350	Palmitic acid content
	19	47120000	49070000	18898	47922295	48033350	Marbling score
	21	14830000	16650000	152013	14912061	14912101	Kidney, pelvic, and heart fat percentage
	21	14830000	16650000	155897	15048672	15048712	Milk tridecylic acid content
	21	14830000	16650000	18768	15078554	15238690	Rump width
	21	14830000	16650000	96526	15281462	15281502	Bovine tuberculosis susceptibility
	21	14830000	16650000	131257	16075251	16075291	Dry matter intake
	21	14830000	16650000	165557	16590863	16590903	Milk lactoperoxidase content
IND/NorthAFT comparison (*Rsb* and *XP-EHH*)	12	28400000	30490000	15277	29036242	29036282	Inseminations per conception
	18	11580000	14350000	155610	11616263	11616303	Milk tridecylic acid content
	18	11580000	14350000	175782	13411141	13411181	Somatic cell score
	18	11580000	14350000	167831	14688573	14688613	Bovine tuberculosis susceptibility

Supplementary Table S3. List of previously reported bovine structural variants (SV) within the candidate region intervals identified by at least two EHH-based approaches.

BTA	Interval (bp)	SV ID	SV Type	SV start (bp)	SV end (bp)	SV length (Mb)
1	17,700,000-19,640,000	esv4012563	CNV	17782548	17787780	0.005232
		esv4013690	CNV	17939173	17940403	0.00123
		esv3897495	inversion	17940520	17949579	0.009059
		esv4017767	CNV	17973227	17973227	0
		esv4019623	CNV	18024600	18024821	0.000221
		esv3897502	inversion	18146776	18155461	0.008685
		esv3897501	inversion	18147300	18154930	0.00763
		esv3899600	tandem_duplication	18280913	18285129	0.004216
		esv4014647	CNV	18324099	18328643	0.004544
		esv4012014	CNV	18358816	18359746	0.00093
		esv4017855	CNV	18422681	18422920	0.000239
		esv4017405	CNV	18530763	18537550	0.006787
		nsv810736	CNV	18561545	18679089	0.117544
		nsv835049	CNV	18636480	18649420	0.01294
		esv4018546	CNV	18787130	18787548	0.000418
		esv3897503	inversion	18835764	18837587	0.001823
		esv3897496	inversion	18949213	18950392	0.001179
		esv3894497	CNV	19191322	19191389	6.7E-05
		esv4015306	CNV	19303404	19303951	0.000547
		esv3894499	CNV	19523514	19523639	0.000125
4	76,570,000-78,910,000	nsv835587	CNV	76880645	76899028	0.01838
		esv4017615	CNV	76924883	76925200	0.00032

		esv4016541	CNV	77155493	77155697	0.00020
		esv3895676	CNV	77855433	77855517	0.00008
		esv4016848	CNV	78221377	78222882	0.00151
		nsv834779	CNV	78540300	78562754	0.02245
		esv4016890	CNV	78900615	78900849	0.00023
4	113,060,000-114,940,000	nsv616158	CNV	112828305	113396585	0.56828
		nsv809903	CNV	112842657	113396585	0.55393
		nsv811070	CNV	112920885	113396585	0.47570
		esv4018773	CNV	113061203	113065858	0.00466
		esv3898436	inversion	113073049	113234613	0.16156
		nsv810805	CNV	113089386	113209434	0.12005
		nsv2727653	CNV	113118864	113200469	0.08161
		nsv2727657	CNV	113129045	113139001	0.00996
		nsv2727624	CNV	113153981	113205825	0.05184
		esv4017462	CNV	113162621	113163663	0.00104
		esv4013431	CNV	113165632	113166716	0.00108
		esv4018935	CNV	113187660	113188854	0.00119
		esv4014938	CNV	113194108	113196088	0.00198
		nsv834804	CNV	113271539	113396585	0.12505
		nsv835796	CNV	113312906	113352188	0.03928
		esv3898437	inversion	113337523	113350136	0.01261
		esv4017582	CNV	113507935	113510556	0.00262
		esv4012092	CNV	113948479	113948823	0.00034
		esv4011978	CNV	113948630	113948971	0.00034

		esv3898443	inversion	114342437	114343869	0.00143
		nsv810806	CNV	114602925	114669240	0.06632
		esv4019421	CNV	114641443	114641685	0.00024
6	46,780,000-50,050,000	esv4012623	CNV	46788133	46792129	0.00400
		esv4015844	CNV	46920742	46920744	0.00000
		esv3895963	CNV	46943071	46943072	0.00000
		esv4014167	CNV	47194215	47194474	0.00026
		esv4018790	CNV	47220158	47220162	0.00000
		esv4011778	CNV	47268615	47268876	0.00026
		esv4015635	CNV	47376204	47379449	0.00325
		nsv809962	CNV	47488681	47602962	0.11428
		nsv810831	CNV	47488681	47602962	0.11428
		esv4011288	CNV	47513134	47516523	0.00339
		nsv2727734	CNV	47585179	47592839	0.00766
		esv4018080	CNV	47592134	47592139	0.00001
		esv4013229	CNV	47592278	47592287	0.00001
		esv4015246	CNV	47636584	47637385	0.00080
		esv4018906	CNV	47681935	47690725	0.00879
		esv4016520	CNV	47759149	47759638	0.00049
		nsv2727735	CNV	48005385	48018440	0.01306
		esv4012430	CNV	48009168	48009171	0.00000
		esv3895965	CNV	48011404	48011407	0.00000
		esv4015494	CNV	48077419	54826474	6.74906
		esv4016472	CNV	48182377	48183185	0.00081

		esv4019143	CNV	48285495	48290711	0.00522
		esv4014390	CNV	48328670	48328987	0.00032
		esv4016647	CNV	48404580	48404869	0.00029
		esv4012945	CNV	48537124	48733531	0.19641
		nsv834879	CNV	48640022	48665947	0.02593
		esv4014742	CNV	48731333	48731339	0.00001
		esv4016453	CNV	48745327	48745327	0.00000
		esv4017595	CNV	48945472	48946237	0.00077
		nsv811093	CNV	49416370	49503645	0.08728
		esv4012249	CNV	49471457	49471460	0.00000
		esv4018113	CNV	49710295	49710872	0.00058
		esv4014615	CNV	49823289	49824450	0.00116
		esv4012314	CNV	49910321	49912371	0.00205
		esv4014364	CNV	49945832	49946551	0.00072
		esv4012989	CNV	49956525	49956533	0.00001
7	41,060,000-43,620,000	esv4018375	CNV	41249044	41251414	0.00237
		esv3896137	CNV	41253325	41279863	0.02654
		esv3896124	CNV	41364968	41365056	0.00009
		esv4013684	CNV	41700594	41701996	0.00140
		esv4012898	CNV	41703058	41703848	0.00079
		nsv2779243	CNV	41748862	42002503	0.25364
		esv3898709	inversion	41830769	41832559	0.00179
		nsv835659	CNV	41861186	41895576	0.03439
		esv4017799	CNV	41908923	41948069	0.03915

		nsv835178	CNV	41915155	41931890	0.01674
		esv4016128	CNV	41983975	41984902	0.00093
		nsv2727794	CNV	42154844	42170251	0.01541
		nsv2727810	CNV	42180257	42191782	0.01153
		esv4011530	CNV	42194921	42206111	0.01119
		esv4017866	CNV	42231407	42231780	0.00037
		nsv835963	CNV	42326751	42356881	0.03013
		esv4016899	CNV	42368758	42369295	0.00054
		esv4013136	CNV	42379968	42380308	0.00034
		nsv2727829	CNV	42494001	42495839	0.00184
		esv4011575	CNV	42525058	42528380	0.00332
		esv4014280	CNV	42659351	42659687	0.00034
		nsv2727813	CNV	42701558	42726386	0.02483
		nsv835310	CNV	42890840	42949882	0.05904
		esv3900095	tandem_duplication	42941250	42941259	0.00001
		esv3900096	tandem_duplication	42941250	42941273	0.00002
		esv3896140	CNV	42944532	42944919	0.00039
		nsv2779244	CNV	43194382	43278613	0.08423
		nsv809996	CNV	43482925	43792867	0.30994
12	28,400,000-30,490,000	esv4012356	CNV	23433844	29409766	5.97592
		esv4018299	CNV	29154773	29155553	0.00078
		esv4013483	CNV	30344074	30350397	0.00632
18	11,580,000-14,350,000	nsv2728434	CNV	10304394	15180459	4.87607
		esv4014777	CNV	11979106	11980300	0.00119

		nsv810275	CNV	12358306	12450150	0.09184
		esv3894276	CNV	13234939	13235013	0.00007
		nsv616076	CNV	13311159	13393029	0.08187
		esv3894277	CNV	13718924	13718925	0.00000
19	47,120,000-49,070,000	esv4014820	CNV	47360707	47362353	0.001646
		esv4012518	CNV	48115912	48116810	0.00090
		esv3894457	CNV	48208345	48223552	0.01521
		esv3894458	CNV	48210991	48226204	0.01521
		esv3899581	tandem_duplication	48214855	48245953	0.03110
		nsv835358	CNV	48219399	48263515	0.04412
		esv3899580	tandem_duplication	48225976	48241807	0.01583
		esv3899582	tandem_duplication	48232328	48248185	0.01586
21	14,830,000-16,650,000	nsv810995	CNV	15236214	15335015	0.09880
		esv4017594	CNV	15244984	15245242	0.00026
		nsv810338	CNV	15264312	15335015	0.07070
		esv4012097	CNV	15549061	15549792	0.00073
		esv4018178	CNV	15707133	15709563	0.00243
		esv4019229	CNV	15836149	15837991	0.00184
		esv4012534	CNV	15840080	15841925	0.00185
		esv3894675	CNV	15868997	15869070	0.00007
		nsv835705	CNV	16203062	16215672	0.01261
		nsv2727212	CNV	16472985	17140695	0.66771
		esv4015977	CNV	16560100	16560103	0.00000
24	18,030,000-20,020,000	esv4014153	CNV	18078880	18079580	0.00070

		esv4018650	CNV	18230953	18231567	0.00061
		nsv2727301	CNV	18840515	18853014	0.01250
		esv4014273	CNV	19049432	19050201	0.00077
		esv4019570	CNV	19345540	19346513	0.00097
		esv4013891	CNV	19673406	19675093	0.00169
		esv4019212	CNV	19910089	19910375	0.00029
		esv3897853	inversion	19970607	19983771	0.01316
		esv3897852	inversion	19972990	19978876	0.00589
24	59,750,000-61,740,000	esv4016428	CNV	59928522	59929229	0.00071
		nsv810701	CNV	59946142	60035969	0.08983
		esv4016185	CNV	59947818	59949680	0.00186
		nsv834884	CNV	59995865	60005963	0.01010
		nsv811017	CNV	60035969	60156075	0.12011
		esv3894988	CNV	60231682	60231774	0.00009
		esv3894989	CNV	60284481	60284554	0.00007
		esv4012433	CNV	60306686	60307344	0.00066
		esv3897882	inversion	60409436	60417429	0.00799
		esv4019379	CNV	60995488	60995835	0.00035
		esv3894990	CNV	61261105	61261192	0.00009
		esv3894991	CNV	61278602	61278699	0.00010
		esv3894992	CNV	61453757	61453788	0.00003
		esv4014585	CNV	61558362	61558629	0.00027
		esv3894993	CNV	61600433	61600471	0.00004
		esv4018054	CNV	61731810	61732114	0.00030

Supplementary Table S4. List of genes co-localizing with the bovine CNVs listed above (Supplementary Table S3).

BTA	CNV_ID	CNV Start (bp)	CNV end (bp)	Genes
1	nsv810736	18561545	18679089	*TMPRSS15*
4	nsv835587	76880645	76899028	*NPC1L1*
4	esv4017615	76924883	76925200	*NUDCD3*
4	esv4016890	78900615	78900849	*GLI3*
4	nsv616158	112828305	113396585	*GIMAP4, GIMAP7, GIMAP5*
4	nsv810806	114602925	114669240	*KMT2C*
6	esv4012989	49956525	49956533	*PCDH7*
7	esv3896137	41253325	41279863	*OR2W3*
7	nsv2779243	41748862	42002503	*OR2L13*
7	nsv2727829	42494001	42495839	*MGC137030*
7	esv4011575	42525058	42528380	*LYPD8*
7	nsv2727813	42701558	42726386	*PGBD2*
7	nsv2779244	43194382	43278613	*BSG, HCN2, POLRMT, FGF22, RNF126*
7	nsv809996	43482925	43792867	*WDR18, GRIN3B, TMEM259, CNN2, ARHGAP45, POLR2E, GPX4, SBNO2*
12	esv4012356	23433844	29409766	*FRY, RXFP2*
18	nsv2728434	10304394	15180459	*GSE1, GINS2, EMC8, COX4I1, IRF8, FOXF1, MTHFSD, FOXC2, FOXL1, FBXO31, MAP1LC3B, ZCCHC14, JPH3, KLHDC4, SLC7A5, CA5A, BANP, ZFPM1, ZC3H18, CYBA, MVD, SNAI3, CTU2, RNF166, PIEZO1, CDT1, APRT, GALNS, TRAPPC2L, CBFA2T3, ACSF3, CDH15, SLC22A31*
21	esv4012534	15840080	15841925	*SV2B*
21	nsv835705	16203062	16215672	*AKAP13*
21	nsv2727212	16472985	17140695	*KLHL25*

24	esv4013891	19673406	19675093	*CELF4*
24	nsv810701	59946142	60035969	*CDH20*
24	esv3894990	61261105	61261192	*PHLPP1*
24	esv4014585	61558362	61558629	*BCL2*
24	esv3894993	61600433	61600471	*KDSR*
24	esv4018054	61731810	61732114	*SERPINB5*

Supplementary Table S5. Results of Bayescan analysis showing outlier SNPs found between North African and African breeds. SNPs in bold are located near or within candidate regions identified either by the *Rsb* or *XP-EHH* statistic.

BTA	SNP name	position	qval	Fst	Gene
1	ARSBFGLNGS11433	2574098	0.013527	0.23802	
1	BTB00016647	33342088	0.000920	0.28188	CADM2
1	BTA89822nors	45869465	0.0041827	0.27595	SENP7
1	ARSBFGLNGS102593	147189105	0.03864	0.22926	RUNX1
1	Hapmap48261BTA24663	150888684	0.017524	0.25746	ERG
1	BTB01585499	157367219	0.011676	0.26414	KAT2B
2	ARSBFGLNGS117095	60165124	0.0098373	0.26503	THSD7B
2	ARSBFGLNGS105413	135305397	0.025468	0.25256	
3	ARSBFGLNGS38423	12671675	0.0015336	0.28004	FCRL3
3	ARSBFGLNGS2988	115090121	0.040891	0.24817	AGAP1
**4**	**Hapmap47085BTA72511**	**112769671**	**0.029584**	**0.22956**	
5	ARSBFGLNGS4312	26750096	0.046731	0.24304	HDAC9
5	Hapmap23876BTA143610	111853277	0.030671	0.25274	MKL1
6	BTA121695nors	90810672	0.0088768	0.2656	SDAD1
6	BTB00275668	101294671	0.02434	0.25441	MAPK10
8	ARSBFGLNGS765	27285102	0.0020004	0.28165	
9	BTB00385217	24658856	0.043025	0.2239	
9	ARSBFGLNGS111000	51148597	0.018611	0.25768	
9	Hapmap51986BTA84173	71322875	0.041974	0.24734	
9	Hapmap42338BTA84177	71493970	0.046731	0.24407	EYA4
11	ARSBFGLNGS57976	9303545	0.012613	0.26379	FHL2
11	ARSBFGLNGS20828	25341825	0.039781	0.22668	
11	UAIFASA8854	49640162	1E-05	0.31709	ELMOD3
11	ARSBFGLNGS110881	68147744	0.005201	0.26715	ANXA4
11	ARSBFGLNGS18890	88733426	0.015412	0.25825	
12	ARSBFGLNGS18439	84745020	0.04427	0.24426	
13	BTA92225nors	460596	0.033877	0.22831	
14	UAIFASA1789	32282448	0.029584	0.25227	
14	ARSBFGLNGS33213	66308321	0.00693	0.27058	MATN2
15	ARSBFGLNGS102526	82933219	0.023191	0.25406	
16	ARSBFGLNGS84156	42063543	0.015412	0.26041	
16	Hapmap42930BTA39624	64188174	0.0061243	0.26752	
16	ARSBFGLNGS15423	72258249	0.00020004	0.29917	KCNH1
16	ARSBFGLNGS17566	74876995	0.035002	0.25039	

17	Hapmap57796rs29012758	59899411	0.0078816	0.2682	LOC107131338
17	ARSBFGLNGS119720	60109941	0.023191	0.25432	
18	ARSBFGLNGS32712	11499360	0.031727	0.25194	GSE1
18	ARSBFGLNGS101491	13794268	0.0034407	0.27174	
19	ARSBFGLNGS112120	30101489	0.019671	0.25654	SHISA6
19	ARSBFGLNGS68861	50539250	0.032735	0.25069	LOC781977
19	ARSBFGLNGS28682	58908149	0.026551	0.25315	
21	BTB01714745	9865536	1E-05	0.31414	
21	ARSBFGLNGS117259	69165607	0.037459	0.2499	
22	ARSBFGLNGS114883	19403914	0.020761	0.25708	GRM7
22	Hapmap47321BTA53898	24557949	0.0023755	0.28128	
22	ARSBFGLNGS39459	49904097	0.047898	0.24541	CACNA2D2
23	ARSBFGLNGS16284	24922231	6.67E-05	0.30531	TRAM2
23	ARSBFGLNGS97912	40543523	0.0027339	0.27951	ATXN1
23	BTB01174913	48550246	0.036241	0.25123	LY86
24	ARSBFGLNGS41126	60875114	0.016412	0.23377	
25	ARSBFGLNGS13398	40240409	0.010724	0.2644	
28	ARSBFGLNGS12759	15620196	0.049089	0.2434	
29	ARSBFGLNGS118114	29629712	0.027594	0.25516	TIRAP

Supplementary Table S6. Results of Bayescan analysis showing outlier SNPs found between North African and European breeds. SNPs in bold are located near or within candidate regions identified either by the *Rsb* or *XP-EHH* statistic.

BTA	SNP name	position	qval	Fst	Gene
1	ARSBFGLNGS78179	15574241	0.020512	0.19465	NCAM2
1	ARSBFGLNGS106432	42264448	0.011767	0.20274	GABRR3
1	BTB01462011	45581665	0.029973	0.18844	
2	ARSBFGLNGS100793	89217187	0.013992	0.20427	
3	INRA170	52783346	0.017858	0.23094	
4	ARSBFGLNGS66729	51914423	0.000440	0.23028	LOC100140425
5	BTA72821nors	18367350	0.036159	0.18837	
5	ARSBFGLNGS92172	50617288	0.012925	0.23624	
5	Hapmap40583BTA74092	74547557	0.0044809	0.21305	LOC100847379
6	ARSBFGLNGS100658	57842883	0.015293	0.20226	
6	ARSBFGLNGS83066	91223593	0.0081874	0.20462	STBD1
**7**	**ARSBFGLNGS36580**	**37529456**	**0.0058845**	**0.21984**	
**7**	**ARSBFGLNGS83376**	**44379268**	**0.0036452**	**0.21979**	
**7**	**ARSBFGLNGS81015**	**46940783**	**0.010602**	**0.20505**	
8	ARSBFGLNGS108557	72603540	0.0011002	0.22412	DOCK5
8	Hapmap43489BTA121857	73116327	0.0027756	0.21864	
**8**	**BTA82340nors**	**88899293**	**0.026577**	**0.19146**	
11	BTA119859nors	28216046	0.031813	0.19237	PRKCE
11	UAIFASA8854	49640162	1.02E-05	0.34381	ELMOD3
11	ARSBFGLNGS42014	87403249	0.046778	0.18403	
13	BTA32929nors	8018225	0.01916	0.19776	MACROD2
13	ARSBFGLBAC11941	31459180	0.025109	0.19613	CUBN
15	Hapmap50533BTA99607	40783003	0.040534	0.18396	USP47
15	ARSBFGLNGS113600	73619449	0.042703	0.18011	
16	ARSBFGLNGS42691	23317276	0.0070476	0.20791	
16	Hapmap42930BTA39624	64188174	0.0094152	0.24754	
16	BTB00654719	66726004	0.038378	0.21062	HMCN1
16	ARSBFGLNGS15423	72258249	0.000102	0.3068	KCNH1
17	ARSBFGLNGS102063	34169704	0.023559	0.19225	
19	ARSBFGLNGS103353	24974201	0.016527	0.19832	SPNS2
19	ARSBFGLNGS106336	36107322	0.028254	0.18996	CACNA1G
21	BTB01714745	9865536	1E-05	0.3406	
21	ARSBFGLNGS75411	63079093	0.021996	0.19624	

21	Hapmap41013BTA52932	63717522	0.0019432	0.21686	
23	ARSBFGLNGS16284	24922231	0.0001002	0.31569	TRAM2
24	BTA42867nors	48949533	0.048676	0.17967	DYM2
26	BTA111275nors	9161929	0.044793	0.18144	MINPP1
27	BTA66980nors	23109828	0.005201	0.23439	
28	ARSBFGLNGS107963	26627985	0.033901	0.18726	

Supplementary Table S7. Functional annotation clustering results for candidate genes jointly identified by Rsb and XP-EHH tests for the AFT/North African comparison following DAVID analysis. Significantly enriched functional term clusters (Benjamin-corrected p-value < 0.05) are in bold.

**Annotation Cluster 1**	**Enrichment Score: 5.468314**				
**Category**	**Term**	**Count**	**PValue**	**Genes**	**Benjamini**
**INTERPRO**	**IPR006703:AIG1**	**6**	**2.11E-09**	**ENSBTAG00000037510, ENSBTAG00000008550, ENSBTAG00000004894, ENSBTAG00000040331, ENSBTAG00000030940, ENSBTAG00000000715**	**4.45E-07**
**INTERPRO**	**IPR027417:P-loop containing nucleoside triphosphate hydrolase**	**14**	**2.94E-05**	**ENSBTAG00000018838, ENSBTAG00000037510, ENSBTAG00000008550, ENSBTAG00000010602, ENSBTAG00000040331, ENSBTAG00000008468, ENSBTAG00000004894, ENSBTAG00000006377, ENSBTAG00000007759, ENSBTAG00000000607, ENSBTAG00000030940, ENSBTAG00000000715, ENSBTAG00000010492, ENSBTAG00000031861**	**0.003105**
**GOTERM_MF_DIRECT**	**GO:0005525~GTP binding**	**8**	**6.34E-04**	**ENSBTAG00000037510, ENSBTAG00000008550, ENSBTAG00000004894, ENSBTAG00000040331, ENSBTAG00000008468, ENSBTAG00000030940, ENSBTAG00000000715, ENSBTAG00000031861**	**0.068437**
Annotation Cluster 2	Enrichment Score: 1.555891				
Category	Term	Count	PValue	Genes	Benjamini
UP_KEYWORDS	ATP-binding	9	0.017811	ENSBTAG00000012653, ENSBTAG00000008143, ENSBTAG00000007759, ENSBTAG00000010602, ENSBTAG00000000607, ENSBTAG00000007766, ENSBTAG00000010492, ENSBTAG00000032288, ENSBTAG00000006377	0.942465
UP_KEYWORDS	Nucleotide-binding	10	0.031349	ENSBTAG00000012653, ENSBTAG00000008143, ENSBTAG00000007759, ENSBTAG00000010602, ENSBTAG00000000607, ENSBTAG00000007766, ENSBTAG00000010492, ENSBTAG00000032288, ENSBTAG00000031861, ENSBTAG00000006377	0.942465
UP_SEQ_FEATURE	nucleotide phosphate-binding region:ATP	4	0.032357	ENSBTAG00000012653, ENSBTAG00000010602, ENSBTAG00000007766, ENSBTAG00000010492	0.931096
GOTERM_MF_DIRECT	GO:0005524~ATP binding	11	0.03307	ENSBTAG00000012653, ENSBTAG00000018838, ENSBTAG00000008143, ENSBTAG00000007759, ENSBTAG00000010602, ENSBTAG00000002917, ENSBTAG00000000607, ENSBTAG00000007766, ENSBTAG00000010492, ENSBTAG00000032288, ENSBTAG00000006377	1
Annotation Cluster 3	Enrichment Score: 1.475098				

Category	Term	Count	PValue	Genes	Benjamini
UP_SEQ_FEATURE	lipid moiety-binding region:S-palmitoyl cysteine	3	0.016168	ENSBTAG00000004869, ENSBTAG00000017680, ENSBTAG00000000274	0.931096
UP_KEYWORDS	Lipoprotein	5	0.033361	ENSBTAG00000004869, ENSBTAG00000000597, ENSBTAG00000017680, ENSBTAG00000031861, ENSBTAG00000000274	0.942465
UP_KEYWORDS	Palmitate	3	0.069628	ENSBTAG00000004869, ENSBTAG00000017680, ENSBTAG00000000274	0.989659
Annotation Cluster 4	Enrichment Score: 0.821316				
Category	Term	Count	PValue	Genes	Benjamini
UP_KEYWORDS	Membrane	30	0.054999	ENSBTAG00000012653, ENSBTAG00000037510, ENSBTAG00000011226, ENSBTAG00000010612, ENSBTAG00000011229, ENSBTAG00000045535, ENSBTAG00000007569, ENSBTAG00000007723, ENSBTAG00000007766, ENSBTAG00000044146, ENSBTAG00000007762, ENSBTAG00000000274, ENSBTAG00000000597, ENSBTAG00000021260, ENSBTAG00000017680, ENSBTAG00000012031, ENSBTAG00000000715, ENSBTAG00000010492, ENSBTAG00000012078, ENSBTAG00000019302, ENSBTAG00000016766, ENSBTAG00000012968, ENSBTAG00000040331, ENSBTAG00000000061, ENSBTAG00000004869, ENSBTAG00000000588, ENSBTAG00000007759, ENSBTAG00000001739, ENSBTAG00000031861, ENSBTAG00000016663	0.989659
UP_KEYWORDS	Transmembrane helix	24	0.201576	ENSBTAG00000037510, ENSBTAG00000016766, ENSBTAG00000011226, ENSBTAG00000010612, ENSBTAG00000011229, ENSBTAG00000045535, ENSBTAG00000040331, ENSBTAG00000012968, ENSBTAG00000007569, ENSBTAG00000007723, ENSBTAG00000000061, ENSBTAG00000044146, ENSBTAG00000007762, ENSBTAG00000004869, ENSBTAG00000000588, ENSBTAG00000000597, ENSBTAG00000021260, ENSBTAG00000007759, ENSBTAG00000001739, ENSBTAG00000012031, ENSBTAG00000000715, ENSBTAG00000016663, ENSBTAG00000012078, ENSBTAG00000019302	1
UP_KEYWORDS	Transmembrane	24	0.207045	ENSBTAG00000037510, ENSBTAG00000016766, ENSBTAG00000011226, ENSBTAG00000010612, ENSBTAG00000011229, ENSBTAG00000045535, ENSBTAG00000040331, ENSBTAG00000012968, ENSBTAG00000007569, ENSBTAG00000007723, ENSBTAG00000000061, ENSBTAG00000044146, ENSBTAG00000007762, ENSBTAG00000004869, ENSBTAG00000000588, ENSBTAG00000000597, ENSBTAG00000021260, ENSBTAG00000007759, ENSBTAG00000001739, ENSBTAG00000012031, ENSBTAG00000000715, ENSBTAG00000016663, ENSBTAG00000012078, ENSBTAG00000019302	1

Annotation Cluster 5	Enrichment Score: 0.472306				
Category	Term	Count	PValue	Genes	Benjamini
UP_KEYWORDS	Kinase	5	0.102104	ENSBTAG00000012653, ENSBTAG00000008143, ENSBTAG00000011228, ENSBTAG00000007766, ENSBTAG00000032288	1
UP_KEYWORDS	Serine/threonine-protein kinase	3	0.205608	ENSBTAG00000012653, ENSBTAG00000008143, ENSBTAG00000007766	1
INTERPRO	IPR008271:Serine/threonine-protein kinase, active site	3	0.342645	ENSBTAG00000012653, ENSBTAG00000008143, ENSBTAG00000007766	1
INTERPRO	IPR017441:Protein kinase, ATP binding site	3	0.427151	ENSBTAG00000012653, ENSBTAG00000008143, ENSBTAG00000007766	1
SMART	SM00220:S_TKc	3	0.448627	ENSBTAG00000012653, ENSBTAG00000008143, ENSBTAG00000007766	1
INTERPRO	IPR000719:Protein kinase, catalytic domain	3	0.577403	ENSBTAG00000012653, ENSBTAG00000008143, ENSBTAG00000007766	1
INTERPRO	IPR011009:Protein kinase-like domain	3	0.620838	ENSBTAG00000012653, ENSBTAG00000008143, ENSBTAG00000007766	1
Annotation Cluster 6	Enrichment Score: 0.057347				
Category	Term	Count	PValue	Genes	Benjamini
UP_SEQ_FEATURE	disulfide bond	3	0.738138	ENSBTAG00000004869, ENSBTAG00000012003, ENSBTAG00000000597	1
UP_KEYWORDS	Glycoprotein	3	0.947113	ENSBTAG00000004869, ENSBTAG00000012003, ENSBTAG00000000597	1
UP_KEYWORDS	Disulfide bond	4	0.962540	ENSBTAG00000004869, ENSBTAG00000012003, ENSBTAG00000000597, ENSBTAG00000021260	1

Supplementary Table S8. Functional annotation clustering results for candidate genes jointly identified by *iHS*, *Rsb* and *XP-EHH* tests for the EUT/North African comparison following DAVID analysis. Significantly enriched functional term clusters (Benjamin-corrected p-value < 0.05) are in bold.

Annotation Cluster 1	**Enrichment Score: 6.640408**				
Category	**Term**	**Count**	**PValue**	**Genes**	**Benjamini**
GOTERM_BP_DIRECT	**GO:0007608~sensory perception of smell**	**18**	**1.24E-16**	**ENSBTAG00000009497, ENSBTAG00000009273, ENSBTAG00000047519, ENSBTAG00000025293, ENSBTAG00000046417, ENSBTAG00000001761, ENSBTAG00000046953, ENSBTAG00000003551, ENSBTAG00000045589, ENSBTAG00000021098, ENSBTAG00000048025, ENSBTAG00000046200, ENSBTAG00000030722, ENSBTAG00000030714, ENSBTAG00000016065, ENSBTAG00000013691, ENSBTAG00000039797, ENSBTAG00000034743**	**3.23E-14**
UP_KEYWORDS	**Olfaction**	**21**	**1.21E-09**	**ENSBTAG00000009497, ENSBTAG00000017725, ENSBTAG00000009273, ENSBTAG00000047519, ENSBTAG00000040109, ENSBTAG00000025293, ENSBTAG00000046417, ENSBTAG00000001761, ENSBTAG00000046953, ENSBTAG00000003551, ENSBTAG00000045589, ENSBTAG00000021098, ENSBTAG00000048025, ENSBTAG00000046200, ENSBTAG00000030722, ENSBTAG00000030714, ENSBTAG00000031412, ENSBTAG00000016065, ENSBTAG00000013691, ENSBTAG00000039797, ENSBTAG00000034743**	**1.10E-07**
INTERPRO	**IPR000725: Olfactory receptor**	**21**	**1.71E-09**	**ENSBTAG00000009497, ENSBTAG00000017725, ENSBTAG00000009273, ENSBTAG00000047519, ENSBTAG00000040109, ENSBTAG00000025293, ENSBTAG00000046417, ENSBTAG00000001761, ENSBTAG00000046953, ENSBTAG00000003551, ENSBTAG00000045589, ENSBTAG00000021098, ENSBTAG00000048025, ENSBTAG00000046200, ENSBTAG00000030722, ENSBTAG00000030714, ENSBTAG00000031412, ENSBTAG00000016065, ENSBTAG00000013691, ENSBTAG00000039797, ENSBTAG00000034743**	**2.81E-07**
UP_KEYWORDS	**Sensory transduction**	**21**	**4.58E-09**	**ENSBTAG00000009497, ENSBTAG00000017725, ENSBTAG00000009273, ENSBTAG00000047519, ENSBTAG00000040109, ENSBTAG00000025293, ENSBTAG00000046417, ENSBTAG00000001761, ENSBTAG00000046953, ENSBTAG00000003551, ENSBTAG00000045589, ENSBTAG00000021098, ENSBTAG00000048025, ENSBTAG00000046200, ENSBTAG00000030722, ENSBTAG00000030714, ENSBTAG00000031412, ENSBTAG00000016065, ENSBTAG00000013691, ENSBTAG00000039797, ENSBTAG00000034743**	**2.08E-07**
GOTERM_MF_DIRECT	**GO:0004984~olfactory receptor activity**	**21**	**6.86E-09**	**ENSBTAG00000009497, ENSBTAG00000017725, ENSBTAG00000009273, ENSBTAG00000047519, ENSBTAG00000040109, ENSBTAG00000025293, ENSBTAG00000046417, ENSBTAG00000001761, ENSBTAG00000046953, ENSBTAG00000003551, ENSBTAG00000045589, ENSBTAG00000021098, ENSBTAG00000048025, ENSBTAG00000046200, ENSBTAG00000030722, ENSBTAG00000030714, ENSBTAG00000031412, ENSBTAG00000016065, ENSBTAG00000013691, ENSBTAG00000039797, ENSBTAG00000034743**	**6.66E-07**

GOTERM_BP_DIRECT	**GO:0007186~G-protein coupled receptor signaling pathway**	**19**	**2.67E-08**	**ENSBTAG00000009497, ENSBTAG00000017725, ENSBTAG00000009273, ENSBTAG00000047519, ENSBTAG00000025293, ENSBTAG00000046417, ENSBTAG00000001761, ENSBTAG00000046953, ENSBTAG00000003551, ENSBTAG00000045589, ENSBTAG00000021098, ENSBTAG00000048025, ENSBTAG00000046200, ENSBTAG00000030722, ENSBTAG00000030714, ENSBTAG00000016065, ENSBTAG00000013691, ENSBTAG00000039797, ENSBTAG00000034743**	**3.48E-06**
INTERPRO	**IPR000276: G protein-coupled receptor, rhodopsin-like**	**22**	**5.00E-08**	**ENSBTAG00000009497, ENSBTAG00000017725, ENSBTAG00000009273, ENSBTAG00000047519, ENSBTAG00000040109, ENSBTAG00000025293, ENSBTAG00000046417, ENSBTAG00000001761, ENSBTAG00000046953, ENSBTAG00000003551, ENSBTAG00000045589, ENSBTAG00000021098, ENSBTAG00000048025, ENSBTAG00000046200, ENSBTAG00000030722, ENSBTAG00000030714, ENSBTAG00000031412, ENSBTAG00000037418, ENSBTAG00000016065, ENSBTAG00000013691, ENSBTAG00000039797, ENSBTAG00000034743**	**3.54E-06**
INTERPRO	**IPR017452: GPCR, rhodopsin-like, 7TM**	**22**	**6.48E-08**	**ENSBTAG00000009497, ENSBTAG00000017725, ENSBTAG00000009273, ENSBTAG00000047519, ENSBTAG00000040109, ENSBTAG00000025293, ENSBTAG00000046417, ENSBTAG00000001761, ENSBTAG00000046953, ENSBTAG00000003551, ENSBTAG00000045589, ENSBTAG00000021098, ENSBTAG00000048025, ENSBTAG00000046200, ENSBTAG00000030722, ENSBTAG00000030714, ENSBTAG00000031412, ENSBTAG00000037418, ENSBTAG00000016065, ENSBTAG00000013691, ENSBTAG00000039797, ENSBTAG00000034743**	**3.54E-06**
KEGG_PATHWAY	**bta04740:Olfactory transduction**	**23**	**6.52E-08**	**ENSBTAG00000017725, ENSBTAG00000009273, ENSBTAG00000047519, ENSBTAG00000040109, ENSBTAG00000025293, ENSBTAG00000046417, ENSBTAG00000007557, ENSBTAG00000001761, ENSBTAG00000046953, ENSBTAG00000045644, ENSBTAG00000003551, ENSBTAG00000045589, ENSBTAG00000024653, ENSBTAG00000021098, ENSBTAG00000048025, ENSBTAG00000046200, ENSBTAG00000030722, ENSBTAG00000030714, ENSBTAG00000031412, ENSBTAG00000016065, ENSBTAG00000013691, ENSBTAG00000039797, ENSBTAG00000034743**	**3.46E-06**
GOTERM_MF_DIRECT	**GO:0004930~G-protein coupled receptor activity**	**21**	**1.34E-07**	**ENSBTAG00000009497, ENSBTAG00000017725, ENSBTAG00000009273, ENSBTAG00000047519, ENSBTAG00000040109, ENSBTAG00000025293, ENSBTAG00000046417, ENSBTAG00000001761, ENSBTAG00000046953, ENSBTAG00000003551, ENSBTAG00000045589, ENSBTAG00000021098, ENSBTAG00000048025, ENSBTAG00000046200, ENSBTAG00000030722, ENSBTAG00000030714, ENSBTAG00000031412, ENSBTAG00000016065, ENSBTAG00000013691, ENSBTAG00000039797, ENSBTAG00000034743**	**6.49E-06**
UP_KEYWORDS	**G-protein coupled receptor**	**21**	**1.82E-07**	**ENSBTAG00000009497, ENSBTAG00000017725, ENSBTAG00000009273, ENSBTAG00000047519, ENSBTAG00000040109, ENSBTAG00000025293, ENSBTAG00000046417, ENSBTAG00000001761, ENSBTAG00000046953, ENSBTAG00000003551, ENSBTAG00000045589, ENSBTAG00000021098, ENSBTAG00000048025, ENSBTAG00000046200, ENSBTAG00000030722, ENSBTAG00000030714, ENSBTAG00000031412, ENSBTAG00000016065, ENSBTAG00000013691, ENSBTAG00000039797, ENSBTAG00000034743**	**5.53E-06**

UP_KEYWORDS	**Transducer**	**21**	**3.62E-07**	**ENSBTAG00000009497, ENSBTAG00000017725, ENSBTAG00000009273, ENSBTAG00000047519, ENSBTAG00000040109, ENSBTAG00000025293, ENSBTAG00000046417, ENSBTAG00000001761, ENSBTAG00000046953, ENSBTAG00000003551, ENSBTAG00000045589, ENSBTAG00000021098, ENSBTAG00000048025, ENSBTAG00000046200, ENSBTAG00000030722, ENSBTAG00000030714, ENSBTAG00000031412, ENSBTAG00000016065, ENSBTAG00000013691, ENSBTAG00000039797, ENSBTAG00000034743**	**8.23E-06**
UP_KEYWORDS	**Cell membrane**	**25**	**5.37E-07**	**ENSBTAG00000017725, ENSBTAG00000047519, ENSBTAG00000040109, ENSBTAG00000025293, ENSBTAG00000001761, ENSBTAG00000048025, ENSBTAG00000046200, ENSBTAG00000030722, ENSBTAG00000031412, ENSBTAG00000034743, ENSBTAG00000039797, ENSBTAG00000016648, ENSBTAG00000009497, ENSBTAG00000009273, ENSBTAG00000046417, ENSBTAG00000046953, ENSBTAG00000003551, ENSBTAG00000045589, ENSBTAG00000021098, ENSBTAG00000001652, ENSBTAG00000030711, ENSBTAG00000030714, ENSBTAG00000016065, ENSBTAG00000013691, ENSBTAG00000012066**	**9.78E-06**
UP_KEYWORDS	**Receptor**	**21**	**2.73E-05**	**ENSBTAG00000009497, ENSBTAG00000017725, ENSBTAG00000009273, ENSBTAG00000047519, ENSBTAG00000040109, ENSBTAG00000025293, ENSBTAG00000046417, ENSBTAG00000001761, ENSBTAG00000046953, ENSBTAG00000003551, ENSBTAG00000045589, ENSBTAG00000021098, ENSBTAG00000048025, ENSBTAG00000046200, ENSBTAG00000030722, ENSBTAG00000030714, ENSBTAG00000031412, ENSBTAG00000016065, ENSBTAG00000013691, ENSBTAG00000039797, ENSBTAG00000034743**	**4.13E-04**
UP_KEYWORDS	**Transmembrane helix**	**40**	**7.94E-04**	**ENSBTAG00000017725, ENSBTAG00000016613, ENSBTAG00000047519, ENSBTAG00000040109, ENSBTAG00000025293, ENSBTAG00000027074, ENSBTAG00000044204, ENSBTAG00000020810, ENSBTAG00000001761, ENSBTAG00000004770, ENSBTAG00000007409, ENSBTAG00000048025, ENSBTAG00000046200, ENSBTAG00000030722, ENSBTAG00000031412, ENSBTAG00000024947, ENSBTAG00000000717, ENSBTAG00000037418, ENSBTAG00000039355, ENSBTAG00000034743, ENSBTAG00000039797, ENSBTAG00000016648, ENSBTAG00000009497, ENSBTAG00000009273, ENSBTAG00000020766, ENSBTAG00000046417, ENSBTAG00000046953, ENSBTAG00000003551, ENSBTAG00000045589, ENSBTAG00000024950, ENSBTAG00000021098, ENSBTAG00000001652, ENSBTAG00000004723, ENSBTAG00000030714, ENSBTAG00000015050, ENSBTAG00000016065, ENSBTAG00000013691, ENSBTAG00000012066, ENSBTAG00000011351, ENSBTAG00000038138**	**0.009746**
UP_KEYWORDS	**Transmembrane**	**40**	**8.57E-04**	**ENSBTAG00000017725, ENSBTAG00000016613, ENSBTAG00000047519, ENSBTAG00000040109, ENSBTAG00000025293, ENSBTAG00000027074, ENSBTAG00000044204, ENSBTAG00000020810, ENSBTAG00000001761, ENSBTAG00000004770, ENSBTAG00000007409, ENSBTAG00000048025, ENSBTAG00000046200, ENSBTAG00000030722, ENSBTAG00000031412, ENSBTAG00000024947, ENSBTAG00000000717, ENSBTAG00000037418, ENSBTAG00000039355, ENSBTAG00000034743, ENSBTAG00000039797, ENSBTAG00000016648, ENSBTAG00000009497, ENSBTAG00000009273, ENSBTAG00000020766, ENSBTAG00000046417, ENSBTAG00000046953, ENSBTAG00000003551, ENSBTAG00000045589, ENSBTAG00000024950, ENSBTAG00000021098, ENSBTAG00000001652,**	**0.009746**

				**ENSBTAG00000004723, ENSBTAG00000030714, ENSBTAG00000015050, ENSBTAG00000016065, ENSBTAG00000013691, ENSBTAG00000012066, ENSBTAG00000011351, ENSBTAG00000038138**	
UP_KEYWORDS	**Membrane**	**41**	**0.006001**	**ENSBTAG00000017725, ENSBTAG00000016613, ENSBTAG00000047519, ENSBTAG00000040109, ENSBTAG00000025293, ENSBTAG00000027074, ENSBTAG00000044204, ENSBTAG00000020810, ENSBTAG00000001761, ENSBTAG00000004770, ENSBTAG00000007409, ENSBTAG00000048025, ENSBTAG00000046200, ENSBTAG00000030722, ENSBTAG00000031412, ENSBTAG00000024947, ENSBTAG00000000717, ENSBTAG00000037418, ENSBTAG00000039355, ENSBTAG00000034743, ENSBTAG00000039797, ENSBTAG00000016648, ENSBTAG00000009497, ENSBTAG00000009273, ENSBTAG00000020766, ENSBTAG00000046417, ENSBTAG00000046953, ENSBTAG00000003551, ENSBTAG00000045589, ENSBTAG00000024950, ENSBTAG00000021098, ENSBTAG00000001652, ENSBTAG00000030711, ENSBTAG00000004723, ENSBTAG00000030714, ENSBTAG00000015050, ENSBTAG00000016065, ENSBTAG00000013691, ENSBTAG00000012066, ENSBTAG00000011351, ENSBTAG00000038138**	**0.060677**
Annotation Cluster 2	**Enrichment Score: 2.44265**				
Category	**Term**	**Count**	**PValue**	**Genes**	**Benjamini**
INTERPRO	**IPR001314: Peptidase S1A, chymotrypsin-type**	**6**	**1.93E-04**	**ENSBTAG00000045829, ENSBTAG00000046188, ENSBTAG00000021616, ENSBTAG00000048122, ENSBTAG00000002100, ENSBTAG00000046105**	**0.007914**
SMART	**SM00020:Tryp_SPc**	**6**	**2.30E-04**	**ENSBTAG00000045829, ENSBTAG00000046188, ENSBTAG00000021616, ENSBTAG00000048122, ENSBTAG00000002100, ENSBTAG00000046105**	**0.010367**
INTERPRO	**IPR001254: Peptidase S1**	**6**	**2.96E-04**	**ENSBTAG00000045829, ENSBTAG00000046188, ENSBTAG00000021616, ENSBTAG00000048122, ENSBTAG00000002100, ENSBTAG00000046105**	**0.009711**
INTERPRO	**IPR009003: Trypsin-like cysteine/serine peptidase domain**	**6**	**3.90E-04**	**ENSBTAG00000045829, ENSBTAG00000046188, ENSBTAG00000021616, ENSBTAG00000048122, ENSBTAG00000002100, ENSBTAG00000046105**	**0.010656**
INTERPRO	**IPR018114: Peptidase S1, trypsin**	**5**	**0.001321**	**ENSBTAG00000046188, ENSBTAG00000021616, ENSBTAG00000048122, ENSBTAG00000002100, ENSBTAG00000046105**	**0.030948**

	**family, active site**				
GOTERM_MF_DIRECT	**GO:0004252~serine-type endopeptidase activity**	**6**	**0.001669**	**ENSBTAG00000045829, ENSBTAG00000046188, ENSBTAG00000021616, ENSBTAG00000048122, ENSBTAG00000002100, ENSBTAG00000046105**	**0.053979**
UP_KEYWORDS	**Serine protease**	**4**	**0.015289**	**ENSBTAG00000046188, ENSBTAG00000048122, ENSBTAG00000002100, ENSBTAG00000046105**	**0.139134**
UP_KEYWORDS	**Protease**	**6**	**0.021351**	**ENSBTAG00000046188, ENSBTAG00000024950, ENSBTAG00000048122, ENSBTAG00000002100, ENSBTAG00000046105, ENSBTAG00000038138**	**0.176632**
UP_KEYWORDS	**Hydrolase**	**8**	**0.231271**	**ENSBTAG00000046188, ENSBTAG00000024950, ENSBTAG00000048122, ENSBTAG00000018423, ENSBTAG00000000717, ENSBTAG00000002100, ENSBTAG00000046105, ENSBTAG00000038138**	**1**
UP_KEYWORDS	**Disulfide bond**	**10**	**0.438317**	**ENSBTAG00000046188, ENSBTAG00000016648, ENSBTAG00000003018, ENSBTAG00000001652, ENSBTAG00000021616, ENSBTAG00000048122, ENSBTAG00000017220, ENSBTAG00000012066, ENSBTAG00000002100, ENSBTAG00000046105**	**1**
Annotation Cluster 3	Enrichment Score: 0.777571				
Category	Term	Count	PValue	Genes	Benjamini
INTERPRO	IPR007110:Immunoglobulin-like domain	6	0.072315	ENSBTAG00000016648, ENSBTAG00000007409, ENSBTAG00000024947, ENSBTAG00000044204, ENSBTAG00000039355, ENSBTAG00000012066	1
INTERPRO	IPR013783:Immunoglobulin-like fold	6	0.192550	ENSBTAG00000016648, ENSBTAG00000007409, ENSBTAG00000024947, ENSBTAG00000044204, ENSBTAG00000039355, ENSBTAG00000012066	1
SMART	SM00409:IG	4	0.231929	ENSBTAG00000016648, ENSBTAG00000024947, ENSBTAG00000044204, ENSBTAG00000012066	1
INTERPRO	IPR003599:Immunoglobulin subtype	4	0.240208	ENSBTAG00000016648, ENSBTAG00000024947, ENSBTAG00000044204, ENSBTAG00000012066	1

Annotation Cluster 4	Enrichment Score: 0.562423				
Category	Term	Count	PValue	Genes	Benjamini
INTERPRO	IPR005821:Ion transport domain	3	0.096134	ENSBTAG00000016613, ENSBTAG00000015050, ENSBTAG00000004770	1
UP_KEYWORDS	Ion transport	4	0.299760	ENSBTAG00000016613, ENSBTAG00000001652, ENSBTAG00000015050, ENSBTAG00000004770	1
UP_KEYWORDS	Ion channel	3	0.308474	ENSBTAG00000016613, ENSBTAG00000015050, ENSBTAG00000004770	1
UP_KEYWORDS	Transport	6	0.633050	ENSBTAG00000016613, ENSBTAG00000001652, ENSBTAG00000012225, ENSBTAG00000015050, ENSBTAG00000020810, ENSBTAG00000004770	1
Annotation Cluster 5	Enrichment Score: 0.516235				
Category	Term	Count	PValue	Genes	Benjamini
UP_KEYWORDS	Transcription	7	0.134371	ENSBTAG00000003018, ENSBTAG00000020780, ENSBTAG00000015051, ENSBTAG00000021062, ENSBTAG00000020776, ENSBTAG00000021115, ENSBTAG00000019767	0.794335
GOTERM_BP_DIRECT	GO:0006351~transcription, DNA-templated	6	0.197307	ENSBTAG00000003018, ENSBTAG00000020780, ENSBTAG00000021062, ENSBTAG00000021115, ENSBTAG00000019767, ENSBTAG00000014598	1
UP_KEYWORDS	Transcription regulation	5	0.395771	ENSBTAG00000003018, ENSBTAG00000020780, ENSBTAG00000021062, ENSBTAG00000021115, ENSBTAG00000019767	1
GOTERM_BP_DIRECT	GO:0006355~regulation of transcription, DNA-templated	3	0.820663	ENSBTAG00000021062, ENSBTAG00000021115, ENSBTAG00000019767	1

Annotation Cluster 6	Enrichment Score: 0.436873				
Category	Term	Count	PValue	Genes	Benjamini
UP_KEYWORDS	Zinc	10	0.094409	ENSBTAG00000024950, ENSBTAG00000004637, ENSBTAG00000014349, ENSBTAG00000007470, ENSBTAG00000008154, ENSBTAG00000037383, ENSBTAG00000021115, ENSBTAG00000017220, ENSBTAG00000014598, ENSBTAG00000038138	0.613661
INTERPRO	IPR013083: Zinc finger, RING/FYVE/PHD-type	4	0.318033	ENSBTAG00000004637, ENSBTAG00000014349, ENSBTAG00000007470, ENSBTAG00000014598	1
UP_KEYWORDS	Metal-binding	11	0.382005	ENSBTAG00000024950, ENSBTAG00000004637, ENSBTAG00000014349, ENSBTAG00000007470, ENSBTAG00000008154, ENSBTAG00000037383, ENSBTAG00000020810, ENSBTAG00000021115, ENSBTAG00000017220, ENSBTAG00000014598, ENSBTAG00000038138	1
UP_KEYWORDS	Zinc-finger	5	0.522763	ENSBTAG00000004637, ENSBTAG00000014349, ENSBTAG00000007470, ENSBTAG00000021115, ENSBTAG00000014598	1
GOTERM_MF_DIRECT	GO:0008270~zinc ion binding	6	0.628688	ENSBTAG00000024950, ENSBTAG00000004637, ENSBTAG00000014349, ENSBTAG00000007470, ENSBTAG00000008154, ENSBTAG00000014598	1
GOTERM_MF_DIRECT	GO:0046872~metal ion binding	7	0.634546	ENSBTAG00000024950, ENSBTAG00000037383, ENSBTAG00000020810, ENSBTAG00000021115, ENSBTAG00000017220, ENSBTAG00000046984, ENSBTAG00000038138	1
Annotation Cluster 7	Enrichment Score: 0.407476				
Category	Term	Count	PValue	Genes	Benjamini
SMART	SM00320:WD40	3	0.345247	ENSBTAG00000002434, ENSBTAG00000003500, ENSBTAG00000047217	1
INTERPRO	IPR001680: WD40 repeat	3	0.357688	ENSBTAG00000002434, ENSBTAG00000003500, ENSBTAG00000047217	1
INTERPRO	IPR017986: WD40-	3	0.408710	ENSBTAG00000002434, ENSBTAG00000003500, ENSBTAG00000047217	1

	repeat-containing domain				
INTERPRO	IPR015943: WD40/ YVTN repeat-like-containing domain	3	0.464565	ENSBTAG00000002434, ENSBTAG00000003500, ENSBTAG00000047217	1
Annotation Cluster 8	Enrichment Score: 0.293403				
Category	Term	Count	PValue	Genes	Benjamini
UP_KEYWORDS	Disulfide bond	10	0.438317	ENSBTAG00000046188, ENSBTAG00000016648, ENSBTAG00000003018, ENSBTAG00000001652, ENSBTAG00000021616, ENSBTAG00000048122, ENSBTAG00000017220, ENSBTAG00000012066, ENSBTAG00000002100, ENSBTAG00000046105	1
UP_SEQ_FEATURE	signal peptide	5	0.445528	ENSBTAG00000003018, ENSBTAG00000030711, ENSBTAG00000048122, ENSBTAG00000017220, ENSBTAG00000012066	1
UP_SEQ_FEATURE	disulfide bond	4	0.539684	ENSBTAG00000003018, ENSBTAG00000048122, ENSBTAG00000017220, ENSBTAG00000012066	1
UP_KEYWORDS	Secreted	5	0.636187	ENSBTAG00000024950, ENSBTAG00000003018, ENSBTAG00000030711, ENSBTAG00000048122, ENSBTAG00000017220	1
Annotation Cluster 9	Enrichment Score: 0.17308				
Category	Term	Count	PValue	Genes	Benjamini
UP_KEYWORDS	Nucleus	12	0.428821	ENSBTAG00000003018, ENSBTAG00000014349, ENSBTAG00000002434, ENSBTAG00000021061, ENSBTAG00000021062, ENSBTAG00000021060, ENSBTAG00000045828, ENSBTAG00000020776, ENSBTAG00000021115, ENSBTAG00000019767, ENSBTAG00000046406, ENSBTAG00000000056	1
UP_KEYWORDS	Phosphoprotein	12	0.770141	ENSBTAG00000002645, ENSBTAG00000014349, ENSBTAG00000021061, ENSBTAG00000021062, ENSBTAG00000045828, ENSBTAG00000020810, ENSBTAG00000021115, ENSBTAG00000019767,	1

				ENSBTAG00000017220, ENSBTAG00000020764, ENSBTAG00000012066, ENSBTAG00000000056	
Annotation Cluster 10	Enrichment Score: 0.167121				
Category	Term	Count	PValue	Genes	Benjamini
INTERPRO	IPR027417:P-loop containing nucleoside triphosphate hydrolase	6	0.411645	ENSBTAG00000020780, ENSBTAG00000021061, ENSBTAG00000004249, ENSBTAG00000018423, ENSBTAG00000020766, ENSBTAG00000021058	1
GOTERM_MF_DIRECT	GO:0005524~ATP binding	7	0.701577	ENSBTAG00000021061, ENSBTAG00000008151, ENSBTAG00000002098, ENSBTAG00000018423, ENSBTAG00000020766, ENSBTAG00000021058, ENSBTAG00000000056	1
UP_KEYWORDS	ATP-binding	4	0.802501	ENSBTAG00000021061, ENSBTAG00000002098, ENSBTAG00000018423, ENSBTAG00000020766	1
UP_KEYWORDS	Nucleotide-binding	4	0.925700	ENSBTAG00000021061, ENSBTAG00000002098, ENSBTAG00000018423, ENSBTAG00000020766	1

Supplementary Table S9. Functional annotation clustering results for candidate genes jointly identified by Rsb and XP-EHH tests for the IND/ North African comparison following DAVID analysis.

Category	Term	Count	PValue	Genes	Benjamini
INTERPRO	IPR018122:Transcription factor, fork head, conserved site	3	0.001074	ENSBTAG00000012465, ENSBTAG00000000009, ENSBTAG00000040605	0.100371
INTERPRO	IPR011991:Winged helix-turn-helix DNA-binding domain	5	0.001949	ENSBTAG00000012465, ENSBTAG00000017824, ENSBTAG00000000009, ENSBTAG00000040605, ENSBTAG00000000638	0.100371
INTERPRO	IPR001766:Transcription factor, fork head	3	0.003285	ENSBTAG00000012465, ENSBTAG00000000009, ENSBTAG00000040605	0.112781
SMART	SM00339:FH	3	0.003422	ENSBTAG00000012465, ENSBTAG00000000009, ENSBTAG00000040605	0.071872
UP_KEYWORDS	DNA-binding	5	0.145149	ENSBTAG00000012465, ENSBTAG00000000009, ENSBTAG00000040605, ENSBTAG00000018103, ENSBTAG00000023745	1
Annotation Cluster 2	Enrichment Score: 0.30149401639497786				
Category	Term	Count	PValue	Genes	Benjamini
UP_KEYWORDS	ATP-binding	4	0.362861	ENSBTAG00000012059, ENSBTAG00000009340, ENSBTAG00000015968, ENSBTAG00000005012	1
UP_KEYWORDS	Nucleotide-binding	4	0.546800	ENSBTAG00000012059, ENSBTAG00000009340, ENSBTAG00000015968, ENSBTAG00000005012	1
GOTERM_MF_DIRECT	GO:0005524~ATP binding	4	0.627985	ENSBTAG00000012059, ENSBTAG00000009340, ENSBTAG00000015968, ENSBTAG00000005012	1
Annotation Cluster 3	Enrichment Score: 0.004238555421373555				
Category	Term	Count	PValue	Genes	Benjamini
GOTERM_CC_DIRECT	GO:0016021~integral component of membrane	6	0.985206	ENSBTAG00000011530, ENSBTAG00000006899, ENSBTAG00000020944, ENSBTAG00000016079, ENSBTAG00000015132, ENSBTAG00000013201	1
UP_KEYWORDS	Membrane	9	0.988625	ENSBTAG00000011530, ENSBTAG00000011533, ENSBTAG00000011632, ENSBTAG00000006899, ENSBTAG00000020944, ENSBTAG00000016079, ENSBTAG00000018103, ENSBTAG00000015132, ENSBTAG00000013201	1

UP_KEYWORDS	Transmembrane helix	7	0.993547	ENSBTAG00000011530, ENSBTAG00000011533, ENSBTAG00000006899, ENSBTAG00000020944, ENSBTAG00000016079, ENSBTAG00000015132, ENSBTAG00000013201	1
UP_KEYWORDS	Transmembrane	7	0.993799	ENSBTAG00000011530, ENSBTAG00000011533, ENSBTAG00000006899, ENSBTAG00000020944, ENSBTAG00000016079, ENSBTAG00000015132, ENSBTAG00000013201	1

Supplementary Table S10. Sample description. The fifth column shows the number of individuals after relatedness filtering.

Population name	Abbreviation	Country/continent of origin	Type	Number of individuals	Data origin
Angus	ANG	Europe	EUT	59	Matukumalli et al., 2009
Baladi	BAL	Egypt	North AFT	20	Flori et al., 2018
Biskra	BIS	Algeria	North AFT	22	Flori et al., 2018
Cheurfa	CHE	Algeria	North AFT	27	Flori et al., 2018
Chélifienne	CHF	Algeria	North AFT	28	Flori et al., 2018
Gir	GIR	Asia	IND	22	Matukumalli et al, 2009
Guelmoise	GUE	Algeria	North AFT	27	Flori et al., 2018
Holstein	HOL	Europe	EUT	52	Matukumalli et al., 2009
Jersey	JER	Europe	EUT	26	Matukumalli et al., 2009
Montbeliarde	MON	Europe	EUT	30	Matukumalli et al., 2009
N’dama	ND1	Africa	AFT	14	Gautier et al., 2009
N’dama	ND2	Africa	AFT	17	Gautier et al., 2009
N’dama	NDA	Africa	AFT	23	Matukumalli et al, 2009
Nellore	NEL	Asia	IND	21	Matukumalli et al, 2009
Oulmes Zaer	OUL	Morocco	North AFT	15	Gautier et al., 2009
Tidili	TID	Morocco	North AFT	26	Flori et al., 2018
Brune de l’Atlas	TUNIND	Tunisia	North AFT	39	Ben Jemaa et al., 201
